# Mitochondrial Dysfunction, Neurogenesis, and Epigenetics: Putative Implications for Amyotrophic Lateral Sclerosis Neurodegeneration and Treatment

**DOI:** 10.3389/fnins.2020.00679

**Published:** 2020-07-15

**Authors:** Michele Longoni Calió, Elisandra Henriques, Amanda Siena, Clélia Rejane Antonio Bertoncini, Joana Gil-Mohapel, Tatiana Rosado Rosenstock

**Affiliations:** ^1^Department of Physiology, Federal University of São Paulo, São Paulo, Brazil; ^2^Department of Physiological Science, Santa Casa de São Paulo School of Medical Science, São Paulo, Brazil; ^3^CEDEME, Center of Development of Experimental Models for Medicine and Biology, Federal University of São Paulo, São Paulo, Brazil; ^4^Division of Medical Sciences, Faculty of Medicine, University of Victoria and Island Medical Program, University of British Columbia, Victoria, BC, Canada

**Keywords:** amyotrophic lateral sclerosis, epigenetics, mitochondria, neural stem cells, neurogenesis

## Abstract

Amyotrophic lateral sclerosis (ALS) is a progressive and devastating multifactorial neurodegenerative disorder. Although the pathogenesis of ALS is still not completely understood, numerous studies suggest that mitochondrial deregulation may be implicated in its onset and progression. Interestingly, mitochondrial deregulation has also been associated with changes in neural stem cells (NSC) proliferation, differentiation, and migration. In this review, we highlight the importance of mitochondrial function for neurogenesis, and how both processes are correlated and may contribute to the pathogenesis of ALS; we have focused primarily on preclinical data from animal models of ALS, since to date no studies have evaluated this link using human samples. As there is currently no cure and no effective therapy to counteract ALS, we have also discussed how improving neurogenic function by epigenetic modulation could benefit ALS. In support of this hypothesis, changes in histone deacetylation can alter mitochondrial function, which in turn might ameliorate cellular proliferation as well as neuronal differentiation and migration. We propose that modulation of epigenetics, mitochondrial function, and neurogenesis might provide new hope for ALS patients, and studies exploring these new territories are warranted in the near future.

## Introduction

Amyotrophic lateral sclerosis (ALS), also known as Lou Gherig’s disease, is the third most common adult-onset neurodegenerative disease following Alzheimer’s and Parkinson’s diseases, with 90% of all cases being sporadic (i.e., without known associated genetic cause) (Zarei et al., 2015; Schiaffino et al., 2018). Despite that, mutations in more than two dozen genes have been thought to underlie, at least partially, the neuropathology of both familial ALS (fALS) and sporadic ALS (sALS). Some of the highly penetrant genes include Cu/Zn superoxide dismutase 1 (*SOD1*), Fused in Sarcoma (*FUS*) (4% fALS and < 1% sALS), *C9orf72*, *CHCHD10*, TAR DNA-binding protein 43 (*TDP-43*) (5% fALS and < 1% sALS), and *Sqstm1/p62* (Seetharaman et al., 2009; Blair et al., 2010; Chio et al., 2011; Ludolph et al., 2012; Majounie et al., 2012; Gijselinck et al., 2015; Webster et al., 2016; Carri et al., 2017; Collins and Bowser, 2017; Frick et al., 2018). Indeed, these genetic mutations correspond to ∼68% of fALS cases, while 11% appear to be related to increased susceptibility to sALS (Bozzo et al., 2017; Webster et al., 2018). Mutations in the acetylcholine nicotinic receptors (Sabatelli et al., 2009) and in the charged multivesicular body protein 2b (CHMP2B) (Cox et al., 2010), previously known as chromatin-modifying protein 2b, are also frequent in sALS. However, it is estimated that ∼20% of all fALS cases (and 1% of all sALS cases) are associated with mutations in *SOD1* (Carri et al., 2017). In agreement, more than 170 mutations have now been identified in this gene (Kodavati et al., 2020). However, it is also important to note that recent studies have demonstrated that the most prevalent mutation in ALS seems to be associated with the *C9orf72* gene (40% of fALS cases and 7% of sALS cases) (Carri et al., 2017; Stoccoro et al., 2018). Curiously, more than a hundred low-penetrance ALS loci have been identified, indicating that ALS pathology is also influenced by polygenic inheritance and environmental factors (van Rheenen et al., 2016; Ji et al., 2017; Stoccoro et al., 2018). Thus, ALS can be seen as the outcome of multiple genetic, physiological, and environmental factors, which may contribute to the phenotypic unpredictability associated with both sALS and fALS (Ajroud-Driss and Siddique, 2015).

ALS is characterized by the progressive degeneration of both upper motor neurons in the motor cortex and lower motor neurons in the spinal cord and brainstem (Kunst, 2004; Al-Chalabi et al., 2012; Vandoorne et al., 2018). As a consequence, there is gradual muscle denervation that leads to weakness, atrophy, and paralysis, culminating in lethal respiratory failure (Dupuis et al., 2011; Bucchia et al., 2015). It is reasonable to speculate that the various mechanisms implicated in the pathophysiology of ALS, including the numerous genetic mutations described so far, might affect not only neurons but also non-neuronal cells as well. Indeed, recent studies have shown that astrocytes, oligodendrocytes, and microglia might also play a role in ALS neuropathology (Chen H. et al., 2018). Such findings are reinforced by the presence of several genetic variants in individual ALS patients, suggesting that the interplay among the various mutations may determine disease onset (Cady et al., 2015) and that disease progression and outcomes can be influenced by a variety of factors (Jimenez-Pacheco et al., 2017). Moreover, the non-cell autonomous hypothesis is strengthened by studies involving transcriptome and histology (Chiu et al., 2013; Aronica et al., 2015; Gjoneska et al., 2015; Srinivasan et al., 2016), in which various markers of non-neuronal cells were recognized in induced pluripotent stem cells (iPSCells) from both sALS patients (Re et al., 2014) and SOD1-G93A mice (Nagai et al., 2007; Yamanaka et al., 2008; Ilieva et al., 2009; Chiu et al., 2013; Kang et al., 2013; van Rheenen et al., 2016; Krasemann et al., 2017). Within this scenario, it was also shown that the oligodendrocytic protein myelin-associated oligodendrocyte basic protein (MOBP) was a risk locus for ALS (van Rheenen et al., 2016), and that the expression of the astrocytic protein excitatory amino acid transporter-2 (EAAT2) is reduced and its activity is decreased in the motor cortex and spinal cord of both ALS patients and SOD1G93A transgenic mice (Rothstein et al., 1995, 2005; Ganel et al., 2006; Pardo et al., 2006; Foran et al., 2011; Karki et al., 2015; Lee et al., 2016). Of note, the non-cell autonomous hypothesis has also been corroborated by several *in vitro* studies using cocultures of astrocytes expressing mtSOD1 (G93A) and neurons, cocultures of microglia and neurons, and cultures of motor neurons derived from embryonic stem cells (ESCell) from ALS patients (Di Giorgio et al., 2007; Nagai et al., 2007; Yamanaka et al., 2008; Ferraiuolo et al., 2011; Haidet-Phillips et al., 2011; König et al., 2014; Endo et al., 2015; Johann et al., 2015).

Unfortunately, there is currently no effective treatment or cure for this devastating neurodegenerative disease, albeit some medications used to attenuate symptoms (Brito et al., 2019), such as Rilutek^TM^ (riluzole) and Radicava^TM^ (edaravone) (Martin et al., 1993; Sawada, 2017; Brito et al., 2019). On the other hand, to extend their life expectancy, ALS patients undergo tracheostomy-delivered assisted ventilation (Hayashi and Kato, 1989; Jimenez-Pacheco et al., 2017). Therefore, further elucidation of the neuropathological mechanisms that underlie this disorder is a recognized priority.

Up until a few decades ago, the mammalian brain was believed to be a static organ. However, it is now well established that the brain has the ability to adapt to new and different situations by several mechanisms of synaptic and structural plasticity (jointly referred to as neuroplasticity) that happen well into adulthood. One form of structural neuroplasticity relies on the self-renewal capacity of neural stem cells (NSCs) and neural progenitor cells (NPCs). These cells are now known to reside within restricted brain regions [the subventricular zone (SVZ)/olfactory bulb (OB) and the subgranular zone (SGZ) of the hippocampal dentate gyrus (DG), and a series of sequential events result in the generation of new neurons (Rodriguez and Verkhratsky, 2011a, b)].

In the past decade, several studies have implicated a deregulation of neurogenic function in the mechanisms that result in neurological dysfunction and neurodegeneration (Zhao et al., 2008; Le Grand et al., 2015; Liu and Song, 2016). Indeed, several reports have shown a reduction in neurogenesis in several models of neurodegenerative disorders (Thompson et al., 2008; Marxreiter et al., 2013; Winner and Winkler, 2015; Hollands et al., 2016). In agreement with these observations, various symptoms that are characteristic of the early stages of these neurodegenerative conditions, such as changes in affective behaviors (e.g., anxiety and depression), cognitive deficits, and olfactory disturbances, can be directly related to deregulation of adult neurogenesis. Interestingly, these alterations can be either in the hippocampal DG or in the OB, the two central regions in the adult mammalian brain that retain the capacity to generate new neurons into adulthood (Simuni and Sethi, 2008; Stout et al., 2011; Hinnell et al., 2012). However, these findings seem to vary not only with the brain region, but also with the stage of disease progression (Boekhoorn et al., 2006; Mirochnic et al., 2009) and the species evaluated (Jin et al., 2004; Curtis et al., 2005; Peng et al., 2008; Winner et al., 2008; Kohl et al., 2010; Fedele et al., 2011; Simpson et al., 2011; van den Berge et al., 2011; Calió et al., 2014). Furthermore, these deficits in neurogenic function and affective/cognitive behaviors can both be modulated (i.e., attenuated) by environmental enrichment and physical activity (Chen et al., 2008; Mirochnic et al., 2009; Bossers et al., 2010).

Since the generation of new neurons is directly dependent on cellular energy levels, neurogenesis is considered an adenosine triphosphate (ATP)-dependent mechanism. In support, several studies have revealed the importance of maintaining mitochondrial function for the proliferation of NSCs as well as the survival and differentiation of new neurons (Calingasan et al., 2008; Kirby et al., 2009). In addition, neuronal growth, cytoskeleton remodeling, organelle transport, and the formation and maintenance of synapses also rely on ATP availability (Vayssiere et al., 1992; Bernstein and Bamburg, 2003; Sheng and Cai, 2012).

Considering that a deregulation of adult neurogenesis is a common feature of many neurodegenerative conditions, and taking into account that this is an energy-dependent process, in this mini-review we will discuss the relationship between mitochondrial function and adult neurogenesis in ALS. In addition, we will highlight how epigenetic modulation may be used as a therapeutic strategy to counteract ALS through an improvement of mitochondrial function and a consequent increase in neurogenic rate.

## Mitochondrial Dysfunction in ALS

Mitochondria are essential organelles in eukaryotic cells, whose major function is the production of ATP through oxidative phosphorylation and thus meeting most of the cell’s energy requirements (Nunnari and Suomalainen, 2012; Bernard-Marissal et al., 2018). Of note, several lines of evidence have indicated that the metabolic changes observed in several neurological diseases are the result of a disruption in mitochondrial function and a consequent reduction in ATP production (Herrero-Mendez et al., 2009; Requejo-Aguilar et al., 2014; Camandola and Mattson, 2017; Fiorito et al., 2018). In agreement, a large number of studies have shown that energy metabolism is deregulated in animal models of ALS as well in patients with either sporadic or familial forms of ALS (Bowling et al., 1993; Dupuis et al., 2004, 2011; Browne et al., 2006; Sasaki et al., 2007; Perera and Turner, 2016). Indeed, a decrease in the activity of the electron transport chain (Cozzolino et al., 2009; Crugnola et al., 2010; Kawamata and Manfredi, 2010) and a reduction in the activity of mitochondrial enzymes (Dupuis et al., 2004; De Vos et al., 2007; Gonzalez de Aguilar et al., 2008; Sotelo-Silveira et al., 2009; Bilsland et al., 2010) have been verified to occur in models of this incurable disease. For example, the mSOD1-G93A transgenic mouse model of ALS [which overexpresses the human *SOD1* with the Gly-93-Ala (*G93A*) substitution], has been revealed to display reduced activity of mitochondrial complex I (Jung et al., 2002; Coussee et al., 2011). Interestingly, the discovery of the *G93A* mutation in the antioxidant enzyme SOD1 was the first known genetic cause of human ALS, and ∼160 different mutations affecting the binding of Cu and Zn to the redox center of SOD1 have been identified (Lovejoy and Guillemin, 2014; Malik et al., 2019). There is also much evidence that transition metals, especially Cu, Zn, and Fe, can mediate mitochondrial dysfunction, DNA damage, telomere shortening, and neurodegeneration (Almeida et al., 2006; Lovejoy and Guillemin, 2014; Bertoncini et al., 2016). In ALS patients, magnetic resonance imaging has presented a characteristic T2 shortening, which is attributed to the presence of Fe in the motor cortex. Increased Fe is also detected in the spinal cord of mSOD1 mouse models, and treatment with Fe-chelating drugs lowers levels of Fe in the spinal cord, preserves motor neurons, and extends the lifespan of these animals (Lovejoy and Guillemin, 2014).

Nevertheless, in addition to changes in cellular redox status, alterations in mitochondrial dynamics (Magrane et al., 2012), size (Liu et al., 2013; Wang et al., 2013; Deng et al., 2015; Pansarasa et al., 2018) and localization (Williamson and Cleveland, 1999; Higgins et al., 2002; Magrane et al., 2009, 2012; Zhou et al., 2010; Vande Velde et al., 2011) are also believed to contribute to the pathophysiology of ALS. Indeed, defects in mitochondrial dynamics and disruption of mitochondrial axonal transport have been described in ALS models (De Vos et al., 2007; Shi et al., 2010; Gao et al., 2018). Within this context, Joshi and colleagues have reported excessive mitochondrial fragmentation, mediated by hyperactivation of Drp1, in both fibroblasts derived from numerous forms of fALS and in *SOD1*-mutant motor neurons (Joshi et al., 2018). Accordingly, an improvement in motor performance and an increase in survival were reported in SOD1 G93A mice exposed to a peptide that inhibits the interaction between Drp1 and Fis1 (Joshi et al., 2018). Both *in vivo* studies with the mSOD1-G93A transgenic mouse model and *in vitro* studies with the NSC34 motor neuron cell line (both of which overexpress mSOD1) have described mitochondrial abnormalities as well as altered axonal distribution of these organelles (Williamson and Cleveland, 1999; Magrane et al., 2009, 2012; Vande Velde et al., 2011). Meaningful, mSOD1 tends to accumulate within mitochondria, thus resulting in the accumulation of defective mitochondria (Jaarsma et al., 2001; Pasinelli et al., 2004; Vijayvergiya et al., 2005; Vande Velde et al., 2011; Tafuri et al., 2015). In agreement, studies using patients’ samples have demonstrated the presence of clusters of mitochondria in the anterior region of the lumbar spinal cord (Sasaki and Iwata, 1996) as well as an increase in presynaptic mitochondrial volume in motor neurons (Siklos et al., 1996). Furthermore, mSOD1-G93A transgenic mice exhibited abnormal localization of mitochondria, which may further contribute to mitochondrial dysfunction (Higgins et al., 2002; Zhou et al., 2010). Additionally, Palomo and collaborators have shown that the degradation of dysfunctional mitochondria (i.e., mitophagy) is activated due to the recruitment of the autophagy receptor p62 in the spinal cord of SOD1-G93A mice (Palomo et al., 2018). Furthermore, Miro and Mfn2 (proteins involved in mitochondrial dynamics), Parkin (a ubiquitin ligase), as well as PGC1a (the master regulator of mitochondrial biogenesis) are decreased in these mice (Palomo et al., 2018).

Although most studies that have assessed mitochondrial function in animal models of ALS have primarily used *SOD1* genetic models, it has also been shown that TARDBP, C9orf72, TDP-43, and FUS can also impact this organelle. Fibroblasts with the *TARDBP* (*p.A382T*) mutation present a fragmented mitochondria network as well as changes in mitochondria ultrastructure (Onesto et al., 2016). Moreover, *TARDBP* fibroblasts exhibit a decrease in mitochondrial membrane potential, while *C9orf72* fibroblasts show mitochondrial hyperpolarization as well as an increase in ATP, mitochondrial DNA content, mitochondrial mass, PGC1-α protein, and reactive oxygen species (ROS) levels (Onesto et al., 2016). These results suggest that both *TARDBP* and *C9orf72* mutations can lead to cell death by mechanisms other than RNA metabolism impairment (Onesto et al., 2016). An imbalance between fission and fusion was also observed in *C9orf72* human fibroblasts, as a consequence of increased Mfn1 levels and alterations in mitochondrial shape (Onesto et al., 2016). *C9orf72* was also shown to induce mitochondrial hyperpolarization, in addition to an increase in mitochondrial content and mass, mitochondrial fragmentation, and a loss of mitochondrial cristae (Dafinca et al., 2016; Lopez-Gonzalez et al., 2016). With regard to *TDP-43*, it has been described that the full-length protein can interfere with the mobility of animals (Davis et al., 2018). Curiously, some targets of *TDP-43* include prohibitin 2 (PHB2), a mitochondrial chaperone and mitochondrial degradation receptor, voltage-gated anion channel 1 (VDAC1), and the fusion protein mitofusin 2 (MFN2) (Davis et al., 2018). Furthermore, it has also been shown that the expression of *TDP-43* could lead to the phosphorylation of serine 637 of the DRP1 protein, thus abolishing mitochondrial fission (Davis et al., 2018). Finally, *TDP-43* was also shown to bind to ND3 and ND6 mitochondrial mRNA, thus inhibiting the activity of complex I of the mitochondrial respiratory chain and, consequently, oxidative phosphorylation (Wang et al., 2016). Changes in mitochondrial function have also been documented in *FUS*-associated ALS. Indeed, it has been shown that *FUS* can induce defects in DNA break-ligation mediated by DNA ligase 3 (LIG3), a crucial enzyme for the replication and repair of mtDNA (Kodavati et al., 2020). Furthermore, both R521G and R521H mutations of *FUS* have been associated with smaller mitochondria in motor neurons, deficits in axonal transport, and disruptions in the transference of vesicles between endoplasmic reticulum and mitochondria in iPSC-derived neurons from ALS patients (Tradewell et al., 2012). Moreover, an increase in mitochondrial *FUS* was shown to induce an increase in Fis1 and, as a result, an intensification of mitochondrial fragmentation and ROS production, in addition to mitochondrial depolarization, abnormal mitochondria transport along axons, and a decrease in ATP synthesis (Deng et al., 2020). Together, these studies suggest that unbalanced mitochondrial dynamics may be a common feature in ALS and this can, in turn, lead to a reduction in cell survival.

Though small modifications in the mitochondrial genome can represent a risk factor for neurodegenerative diseases, the sole presence of a few mitochondrial DNA (mtDNA) mutations is not enough to directly lead to neurodegeneration *per se* (Gerschutz et al., 2013; Perier et al., 2013; Parkinson et al., 2014; Smith et al., 2019). Nevertheless, sporadic rearrangements of mtDNA and hereditary mtDNA point mutations have indeed been indirectly linked to neurodegenerative processes (Chinnery and Hudson, 2013; Pinto and Moraes, 2014; Cha et al., 2015; Keogh and Chinnery, 2015; Nissanka and Moraes, 2018). In ALS, in particular, it is known that the amount of mtDNA, a marker for mitochondrial copy number, is reduced in the spinal cord of patients with either familial or sporadic forms of the disease (Wiedemann et al., 2002). Nuclear DNA (nDNA) mutations in genes responsible for mitochondrial proteins have also been connected to ALS (Smith et al., 2019). Taken together, these studies suggest that a decrease in mitochondrial biogenesis may indeed contribute to the pathogenesis of this disorder.

Interestingly, mitochondria are also involved in the buffering of calcium, and a deregulation of mitochondrial-dependent calcium handling has also been linked to neurodegeneration (Rosenstock et al., 2004, 2010a,b; Tang et al., 2005; Damiano et al., 2006; Pelizzoni et al., 2008; Cali et al., 2012; Naia et al., 2014; Carri et al., 2017; Salvatori et al., 2017). Indeed, a disruption of intracellular calcium homeostasis has been revealed to accompany changes in oxidative phosphorylation and ATP synthesis in various neurodegenerative processes, including ALS (Coussee et al., 2011; Ravera et al., 2018).

## Mitochondrial (DYS)Function and Neuroplasticity

**FIGURE 1:**
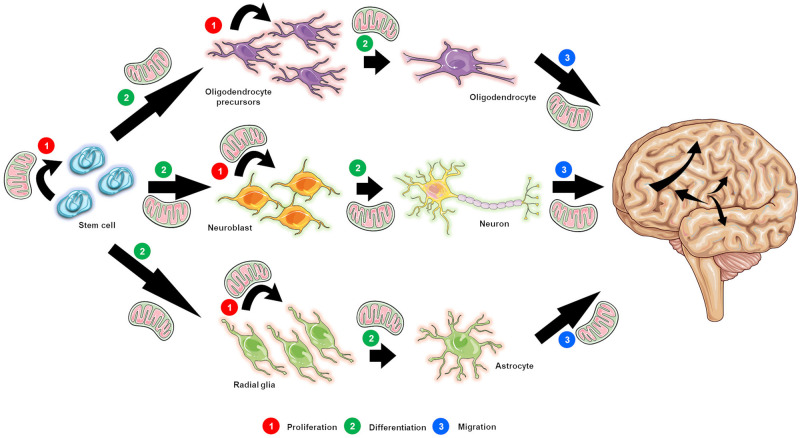
The postulated role of mitochondrial function in neurogenesis. Mitochondrial function is crucial not only for the survival and proliferation of neural stem cells (NSCs; designated as stem cells for simplicity purposes) **(1)**, but also for the proliferation of progenitor and precursor cells **(1)**, and the differentiation **(2)** and migration **(3)** of newly generated neurons. Indeed all of the steps in the neurogenic process are energy dependent, thus relying on intact mitochondrial function.

It is well established that both structural and synaptic neuroplasticity, including cell proliferation, neuronal differentiation, and migration, as well as formation and maintenance of functional synapses, are processes that require energy (i.e., ATP) (Figure 1), and therefore rely on mitochondrial content (Vayssiere et al., 1992; Mattson et al., 2008; Petanjek et al., 2011; Agostini et al., 2016; Lin-Hendel et al., 2016). In support, an increase in the content (i.e., levels) of mitochondrial proteins as well as of transcription factors, which are known to promote an increase in mitochondrial mass, has been detected during early neuronal differentiation (Cordeau-Lossouarn et al., 1991; Vayssiere et al., 1992); these data indicate that mitochondrial content varies during neuronal development. Moreover, in the SVZ niche, mitochondrial genes are the most affected as NSCs progress from quiescent to activated (Mu et al., 2010). In agreement, numerous studies have shown that in addition to requiring appropriate growth factors (Ramasamy et al., 2013) and adequate surfaces (Faissner and Reinhard, 2015), NSCs and their daughter cells undergo various changes concerning their intracellular metabolic machinery in order to proliferate and differentiate (Rafalski and Brunet, 2011; Folmes et al., 2012; Gage and Temple, 2013). Notably, these changes are likely to be correlated with the stage of neuronal differentiation, rather than with simple progression to the postmitotic phase (Vayssiere et al., 1992). Additionally, it has been presented that p53 translocates to mitochondria during the early stages of neuronal differentiation in an attempt to attenuate oxidative stress and decrease mitophagy and cytochrome *c* release, thus contributing to the survival of the newly born neurons and the growth of neuritis, further promoting neuronal differentiation and maturation (Xavier et al., 2013).

Mitochondria are also important for neuronal structure, including axonal and dendritic formation (axonal growth cone and filopodia, respectively); interestingly, both growing axons and dendrites are enriched in these organelles (Cunniff et al., 2016; Han et al., 2016; Sainath et al., 2017; Sheng, 2017; Shneyer et al., 2017; Gershoni-Emek et al., 2018; Smith and Gallo, 2018). Once neurons mature, mitochondrial function (i.e., energy production and supply) is absolutely required for neuronal synaptic plasticity and the formation and maintenance of synapses. Indeed, ATP is necessary for both synaptic vesicle recruitment and neurotransmitter release, as well as the maintenance of ionic and electric gradients across the cell membrane (Tang and Zucker, 1997; Bindokas et al., 1998; Zenisek and Matthews, 2000; Billups and Forsythe, 2002; Kann et al., 2003; Levy et al., 2003; Yang et al., 2003; Verstreken et al., 2005). Consequently, pre- and postsynaptic terminals and nodes of Ranvier have an increased number of mitochondria in comparison to other cellular areas (Fabricius et al., 1993; Li et al., 2004; Zhang et al., 2010).

Noteworthy, disturbances in intracellular calcium buffering by the mitochondria and mitochondrial ROS production can also affect synaptic formation and function. For example, synaptotagmin-1, a protein involved in synaptic vesicle formation, is activated by calcium (Choi, 1985; Schinder et al., 1996; David et al., 1998), and therefore changes in intracellular calcium levels can affect its function. On the other hand, neuronal pruning can be modulated and affected by ROS (Sidlauskaite et al., 2018). Indeed, an increase in mitochondrial ROS production has been observed in inactive synapses, and the presence of ROS in these “weak” synapses may constitute a signal or trigger for their subsequent elimination (Sidlauskaite et al., 2018).

## Changes in Neurogenic Function in ALS

As ALS is a multifactorial disorder (where both genetic predisposition and environmental factors may contribute to its etiology) (Bilsland et al., 2008; Haidet-Phillips et al., 2011; Brites and Vaz, 2014; Nikodemova et al., 2014; Beeldman et al., 2016; Matias-Guiu et al., 2016; Martinez-Merino et al., 2018; Nonneman et al., 2018), changes in neuroplasticity (including altered neurogenic function) may contribute to the pathogenesis of this disease.

To date, various studies have assessed neurogenic function in ALS animal models (Table 1), namely in transgenic mice and rats expressing the mutation in the *SOD1* gene (Warita et al., 2001; Chi et al., 2006, 2007; Liu and Martin, 2006; Murphy, 2009; Li et al., 2012; Khalil and Lievens, 2017). Within this scenario, Li et al. (2012) have reported that mSOD1-G93A rats show a significant reduction in fetal tissue derived NSCs proliferation (Li et al., 2012). In agreement, Liu and Martin (2006) have also observed altered proliferative capacity in all neurogenic niches (SVZ, OB, and hippocampal DG) of the mSOD1-G93A transgenic mouse model. Interestingly, these changes in neurogenic activity can be detected during the presymptomatic phase, before the onset of motor neuron degeneration and subsequent motor paralysis, suggesting that a disruption of the neurogenic process may somewhat contribute to the progression of the disorder in this ALS model (Liu and Martin, 2006). These findings also suggest that ALS-induced alterations in the neurogenic microenvironment (i.e., neurogenic niche) can permanently alter the proliferative capacity of NSCs and NPCs (Lee et al., 2011). Curiously, in 25-week-old mSOD1-G93A transgenic mice, the progressive expression of polysialylated neural cell adhesion molecule (PSA-NCAM, a protein expressed during the maturation and migration of immature neurons and during synaptogenesis) (Rutishauser and Landmesser, 1996; Rutishauser, 2008) has been noted in surviving motor neurons. This outcome suggests that the expression of this protein may dictate, or at least contribute, to the survival of motor neurons in ALS (Warita et al., 2001).

**TABLE 1:** Summary of studies that have assessed adult neurogenesis in ALS animal models.

**Authors (year of publication)**	**Model**	**Main finding**
Warita et al. (2001)	mSOD1-G93A mice	Progressive expression of PSA-NCAM
Liu and Martin (2006)	mSOD1-G93A mice	Alterations in proliferation capacity in SVZ, OB, and DG
Chi et al. (2007)	mSOD1-G93A mice	Augmentation in NSC levels
Lee et al. (2011)	mSOD1-G93A mice	Decrease in functional capacities of neural progenitor cells
Li et al. (2012)	mSOD1-G93A rats	Reduction in NSC proliferation
DiFebo et al. (2012)	Murine model of motor neuron degeneration	Lower NSC proliferation
Marcuzzo et al. (2014)	mSOD1-G93A mice	epSPCs differed more into neurons than into astrocytes
Galán et al. (2017)	Patients	Increase in the neurogenesis in the SVZ, but a decrease of it in the SGZ

NSC proliferation and degeneration of spinal cord motor neurons were also evaluated in a bi-transgenic mouse (Bi-Tg) expressing both mSOD1-G93A and a Nestin enhancer gene (Chi et al., 2007). In this study, Chi and collaborators described an increase in NSC levels in the motor cortex of bi-transgenic animals at the beginning of disease progression when compared with age-matched wild-type controls (Chi et al., 2007). However, as disease progressed, a decrease in NSCs in the lateral ventricles of Bi-Tg was observed, although no changes in the number of NSCs in the hippocampal DG were detected (Chi et al., 2007). Hence, it seems that at least in this model, ALS progression is only related to a decrease in neurogenic function in the SVZ neurogenic niche.

Various *in vitro* studies have also assessed proliferation and differentiation of stem cells derived from animal models of ALS. Marcuzzo et al. (2014) assessed the proliferating and differentiating capacity of ependymal stem progenitors (epSPCs) from the spinal cord of wild-type control, asymptomatic, and symptomatic mSOD1-G93A transgenic mice (Marcuzzo et al., 2014). Surprisingly, these authors found an increase in the number of epSPCs-derived neurons (and a corresponding decrease in the number of epSPCs-derived astrocytes) in mSOD1-G93A transgenic cell populations when compared with wild-type control cells. Oddly, the proportions of oligodendrocytes were similar between both populations. However, G93A-SOD1 epSPCs-derived neurons were smaller than epSPCs-derived wild-type control neurons, whereas G93A-SOD1 epSPCs-derived astrocytes presented an activated phenotype. These marks demonstrate that although SOD1-G93A epSPCs have the potential to differentiate into the three distinct neural linages (neurons, astrocytes, and oligodendrocytes) *in vitro*, the newly generated transgenic cells are morphologically and physiologically different, and such differences might contribute, at least in part, to the neurodegenerative mechanisms underlying this neurological disorder. The neurogenic capacity of SVZ-derived NSCs and its relationship with motor neuron degeneration was also evaluated in the wobbler mouse model, a murine model of motor neuron degeneration characterized by increased cortical hyperexcitability (DiFebo et al., 2012). *In vitro* experiments demonstrated that the rate of wobbler-derived NSC proliferation was significantly lower than in control healthy mice. On the contrary, the number of NSCs exhibiting early neuronal commitment was significantly higher for wobbler-derived NSCs when compared to NSCs from control animals.

So far, only one study has assessed neurogenic capacity in ALS patients. In this study, while an increase in SVZ neurogenesis was observed, a decrease in SGZ neurogenesis was detected in the hippocampal DG of ALS patients (Galán et al., 2017). While the observed increase in SVZ neurogenesis may be part of an endogenous compensatory mechanism to counteract the underlying neurodegenerative process, the real impact of this increase is currently unknown, and future studies are warranted to determine whether the newly generated cells can fully differentiate and migrate toward the areas of ongoing degeneration, or whether they die before becoming fully functional (Galán et al., 2017). Similarly, the impact of the observed reduction in hippocampal DG neurogenesis is unexplained and further investigations are thus necessary to determine its functional implications. Table 1 further summarizes the studies described in this section.

## Regulation of Neurogenic Function in ALS Through Epigenetic Modulation: Possible Therapeutic Avenues

Despite considerable scientific progresses regarding the identification of the molecular underpinnings of ALS pathophysiology, the genesis of this devastating neurological disorder and the factors that dictate its progression remain, for the most part, unknown. As a consequence, no effective treatments are currently available for individuals afflicted with this disease (Mancuso et al., 2014), which makes the search of potential disease-modifying therapies capable of altering the rate of disease progression a recognized priority (Al-Chalabi and Hardiman, 2013). Within this scenario, several lines of evidence have suggested that interventions capable of promoting an increase of neurogenesis may lead to better functional recovery (Marsh and Blurton-Jones, 2017; Kameda et al., 2018; Schiaffino et al., 2018). Indeed, neurogenesis can be modulated by numerous intrinsic and extrinsic factors, including epigenetic modifications, thus suggesting that epigenetic factors may be used as potential targets to promote neurogenic function in models of neurodegeneration. Even though epigenetic modifications are not genetically transmitted, they can be pharmacologically manipulated, making them potential marks for medical intervention (Hwang et al., 2017). In reality, several lines of evidence suggest that epigenetics might not only facilitate the identification of effective therapeutic targets, but also assist with ALS diagnosis and follow-up, since the expression of numerous genes can be modulated by epigenetic mechanisms (Chestnut et al., 2011; Jimenez-Pacheco et al., 2017; Young et al., 2017; Coppedè et al., 2018; Masala et al., 2018).

Epigenetic modifications are mediated through gene–environment interactions (Mehler, 2008; Al-Chalabi and Hardiman, 2013) and result in heritable changes in gene expression that are independent of alterations in DNA sequence (Probst et al., 2009; Bonasio et al., 2010; Rosenstock, 2013). Examples include DNA methylation, posttranslational histone modifications such as methylation, acetylation, phosphorylation, ubiquitination, and isomerization of histones, as well as RNA editing (and non-coding RNA modulation) (Rosenstock, 2013; Bowman and Poirier, 2015; Javaid and Choi, 2017; Jimenez-Pacheco et al., 2017; Koreman et al., 2018; Bennett et al., 2019). Within this scenario, it was demonstrated that miRNA can regulate up to 60% of all protein-coding genes (Friedman et al., 2009). In the context of ALS, it was shown that numerous miRNAs are upregulated, namely miR-155, miR- 22, miR-125b, miR-146b, and miR-365, in SOD1-G93A mice and in the spinal cord of ALS patients (Butovsky et al., 2012; Koval et al., 2013; Parisi et al., 2013, 2016). Of interest, it was also described that the processing and biogenesis of miRNAs can be modified by several proteins, including TDP-43 (Buratti et al., 2005; Kawahara and Mieda-Sato, 2012; Paez-Colasante et al., 2015).

Posttranslational histone modifications can alter the accessibility of DNA to transcription regulators by inducing changes in the structural configuration of nucleosomes (Feng et al., 2015). In particular, histone acetylation is catalyzed by histone acetyltransferases (HATs), and this process results in the loosening of the chromatin structure, which in turn allows for transcriptional activation; on the other hand, histone deacetylases (HDACs) exert the opposite effect (Probst et al., 2009; Feng et al., 2015; Li et al., 2016), and therefore, overexpression of HDACs can have a deleterious effect. In agreement, reduced histone acetylation is a common feature observed in several models of neurodegenerative diseases (Lagali and Picketts, 2011; Naia et al., 2017), and an imbalance between HATs and HDACs activities has been described in ALS (Rouaux et al., 2003; Schmalbach and Petri, 2010). Several *in vivo* studies using ALS animal models and postmortem human tissue have addressed the role of HDACs in modulating disease progression. Of note, an increase in HDAC2 mRNA and a reduction in HDAC11 mRNA (Janssen et al., 2010) has been detected in postmortem spinal cord and brain tissue from ALS patients. Furthermore, disease progression was shown to be associated with an increase in the expression of HDAC4 in muscles in ALS patients (Bruneteau et al., 2013). In agreement, changes in levels of HDACs appear to be correlated with decreased cell death and a delay in disease onset (Rouaux et al., 2003; Yoo and Ko, 2011). On the other hand, preclinical *in vivo* studies with mSOD1-G93A transgenic mice demonstrated that trichostatin A, an inhibitor of HDACs, attenuated motor neuron loss, gliosis, muscular atrophy, and neuromuscular junction denervation, while increasing the survival of transgenic mice (Mancuso et al., 2014). ACY-738, an HDAC inhibitor, was equally able to ameliorate the motor phenotype, spinal cord metabolism, and the life span of a *FUS*-transgenic mouse model (Rossaert et al., 2019). *In vitro* studies have also revealed that inhibition of HDAC class II enhanced the transcription of the glutamate transporter excitatory amino acid transport 2 (EAAT2) and reestablished its expression in *SOD1* animal models (Lapucci et al., 2017). Notably, non-selective HDAC inhibitors can also activate the promoters of the brain-derived neurotrophic factor (*bdnf*) and glial cell line-derived neurotrophic factor (*gdnf*) genes (Wu et al., 2008). Treatment of *SOD1* transgenic animals with a combination of riluzole and an HDAC inhibitor resulted in a 20% increase in survival rate when compared to mice treated with only riluzole, in addition to diminishing the levels of astrogliosis and the death of motor neurons (Del Signore et al., 2009).

The importance of HDACs to ALS is not limited to classic HDACs (classes I, II, and IV) (Gal et al., 2013; Taes et al., 2013; Valle et al., 2014); sirtuins (SIRTs), which are HDACs class III, have also been presented to play an important role in the pathogenesis of this disorder (Giralt and Villarroya, 2012; Rosenstock, 2013; Song et al., 2013; Salvatori et al., 2017). Indeed, studies have demonstrated that Resveratrol (*trans*-3,4’,5-trihydroxystilbene), a natural polyphenol found in grapes, enhanced the enzymatic activity of SIRT1, thus exerting a neuroprotective effect on motor neurons and on muscular fibers (Pasinetti et al., 2013; Song et al., 2014). Furthermore, SIRT1 overexpression in mSOD1-G93A transgenic mice counteracted the toxic effect of mutated *SOD1* in neuronal cultures derived from this transgenic mouse model (Kim et al., 2007). In agreement, administration of resveratrol has also been reported to increase the lifespan of ALS murine models (Douglas and Dillin, 2010; Song et al., 2014), an effect that seems to be related to the expression and activation of several pathways involving not only SIRT1, but also 5’-AMP-activated protein kinase (AMPK) (Mancuso et al., 2014; Song et al., 2014).

Noteworthy, since HDAC can regulate the acetylation of several proteins in addition to histones, numerous pathways other than transcription regulation can be modulated by this class of enzymes (Xiong and Guan, 2012). For example, cellular and, in particular, mitochondrial metabolism can be affected by HDAC activity. Indeed, since ∼99% of all mitochondrial proteins are codified by the nuclear genome, alterations in nuclear DNA triggered by HDAC modulation and epigenetic modifications may in turn affect mitochondrial function (Devall et al., 2016). In support of this hypothesis, it has already been described that SIRT3 (considered the most important deacetylase of mitochondrial proteins) (Lombard et al., 2007) can control not only the levels of mitochondrial phosphorylation, but also the production of ROS and, therefore, levels of oxidative stress (Giralt and Villarroya, 2012) and mitochondrial fragmentation in cortical neurons in the presence of mSOD1 (Song et al., 2013).

Along with mitochondrial function control, it has also been revealed that HDAC modulation regulates autophagy, the main pathway responsible for the degradation of aggregated proteins and deregulated mitochondria (Corcoran et al., 2004; Olzmann et al., 2008; Trüe and Matthias, 2012; Liang et al., 2013). Within this scenario, it has been shown that HDAC1 inhibition induces autophagy (Oh et al., 2008), mitophagy (known to be essential for the maintenance of mitochondrial integrity and function) (Green et al., 2011; Andreux et al., 2013) is increased by overexpression of Sir2 (Koh et al., 2012), and that HDAC6 controls autophagosome and lysosome fusion (Lee et al., 2010). Moreover, in the SOD1-G93A transgenic model of ALS a decrease in HDAC6 expression was found both at the onset and the end stage of disease progression, and the upregulation of this HDAC could increase the life expectancy of these transgenic animals (Chen et al., 2015). However, it was also shown that HDAC6 deficiency could induce an upregulation of tubulin acetylation, which was related to an increase in cell viability (Gal et al., 2013; Taes et al., 2013). More recently, HDAC6 inhibition was suggested to be neuroprotective, since its ablation improves axonal transport and decreases protein aggregation, thus enhancing the clearance of cytosolic proteins (Jimenez-Pacheco et al., 2017). In accordance, Kim and collaborators demonstrated that the TDP-43 and the FUS proteins are able to enclose HDAC6 mRNA (Kim et al., 2010). Given these discrepant results, future studies are warranted to further elucidate the exact role of HDAC6 on the neuropathology of ALS. On the other hand, genetic and pharmacological induction of the mitophagy receptor Nip3-like protein X (NIX) was recently shown to prevent mitochondrial degradation in cells derived from Parkinson’s disease individuals (Koentjoro et al., 2017; Sarkar et al., 2018). Future studies are warranted to determine whether strategy can also be beneficial in models of ALS.

Because epigenetic deregulation may be triggered by the same long-term environmental factors that underlie an increased risk of developing this neurodegenerative disorder, the accumulation of epigenetic modifications throughout life might contribute to the onset and progression of ALS (Paez-Colasante et al., 2015). Indeed, it has been proposed that the silencing of genes that are vital for motor neuron function by epigenetic modifications could underlie, at least in part, sALS. However, several studies revealed an absence of methylation in the promoter region of several ALS-related genes, such as *SOD1*, vascular endothelial growth factor (VEGF), and glutamate type I transporter (GLT1) (Morahan et al., 2007; Oates and Pamphlett, 2007; Yang et al., 2010). However, an increase in DNA methylation was found in the blood of ALS subjects, regardless of the time of onset of the disorder (Tremolizzo et al., 2014). Moreover, total cytosine hydroxymethylation has been found in the brains of end-stage *SOD1* transgenic animals (Figueroa-Romero et al., 2019), while altered levels of DNA methylation have been reported in postmortem brains from sALS individuals when compared to age-matched controls (Morahan et al., 2009). Curiously, 60% of the genes affected by such changes are involved in neurotransmission, oxidative stress, and calcium handling, mechanisms that are thought to be disrupted in ALS (Morahan et al., 2009). Further supporting a role for DNA methylation in ALS is the fact that DNA-(cytosine-5)-methyltransferase 3A (DNMT3A) was shown to be overexpressed in the brain and spinal cord of ALS patients, and this overexpression seems to be related to cell death in motor neuron like cells *in vitro* (Chestnut et al., 2011). In addition, *TDP-43* has been related to uncommon DNA methylation (Appleby-Mallinder et al., 2020). However, methylation of the *C9orf72* gene promoter is still controversial (Gijselinck et al., 2015; Bauer, 2016).

Several other epigenetic alterations have also been described in different cellular and animal models of ALS, including models based on mutations in Sod1 (G93A or H80R), Tardbp, and Fus. These alterations comprise changes in phosphoacetylation of serine 10 and lysine 14 on the H3 tail (H3K14ac-S10ph), dimethylation of lysine 4 on the H3 tail (H3K4me2), and trimethylation of lysine 9 on the H3 tail (H3K9me3) (Jimenez-Pacheco et al., 2017; Masala et al., 2018). It has also been shown that FUS can abrogate histone 4 (H4) methylation in arginine residues by inhibiting methyltransferase PRMT1 (Tibshirani et al., 2015), and that overexpression of human FUS in yeast diminishes H3 acetylation in two different residues, lysine 14 and lysine 56 (H3K14 and H3K56) (Chen K. et al., 2018). In addition, FUS was shown to inhibit CBP/p300 HAT after binding to it, leading to a hypoacetylation state (Alao, 2007; Wang et al., 2008). Of note, the effects of FUS and TDP-43 on epigenome alterations seem to be associated with specific variants of the disease (Masala et al., 2018). In addition, it was recently reported that astrocytes and neurons from C9orf72 BAC mice showed a decrease in H3K9me3 and this was associated with cell death and memory deficiency (Jury et al., 2020).

Given that epigenetics plays a role in the pathogenesis of ALS, we can hypothesize that modulation of neurogenic function through epigenetic modifications may influence disease progression and neurodegeneration in ALS. In support of this hypothesis, recent studies have demonstrated that epigenetic modulation can determine cell type and influence the differentiation of NSCs, during both development and the postnatal period (Chen et al., 2019; Desai et al., 2019). For example, it was verified that moderate changes in the redox status of SIRT1 can suppress NSC proliferation and direct its differentiation toward the astrocytic phenotype, suggesting the existence of a still unidentified metabolic master switch that can determine the fate of neural progenitors (Prozorovski et al., 2008). In addition, inhibition of HDACs is known to prompt neuronal differentiation in NSCs derived from the adult hippocampal DG (Hsieh et al., 2004). Furthermore, valproic acid, a well-known anticonvulsant and mood stabilizer (Henry et al., 2011), was presented to induce neural differentiation of embryonic hippocampal neural progenitor cells *in vitro* and *in vivo* by decreasing proliferation and increasing neuronal differentiation through a mechanism that involves acetylation of histone 3 and 4 (Yu et al., 2009). Valproic acid also seems to inhibit astrocytic and oligodendrocytic differentiation by inducing the expression of neurogenic differentiation factor 1 (NeuroD) (Hsieh et al., 2004).

The role of HAT modulation on neurogenesis is equally promising. It has already been described that a deficiency of HATs reduces the ability of SVZ NSCs to self-renew and differentiate (Merson et al., 2006). For example, an absence of the HAT lysine acetyltransferase 6B (KAT6B) has been shown to result in a reduction in the number of migrating neuroblasts in the rostral migratory stream (RMS) and, consequently, a considerable reduction in the number of new interneurons in the OB (Merson et al., 2006). Increasing evidence has also revealed that histone modification and non-coding RNA expression are closely associated with multiple aspects of the different staged of the adult neurogenesis process (Yao et al., 2016), and histone acetylation in particular is known to affect the differentiation of NSCs (Hsieh et al., 2004; Mu et al., 2010; Kameda et al., 2018).

More recently, a few studies have also assessed whether epigenetic modifications in mtDNA also occur in the context of ALS (Jimenez-Pacheco et al., 2017; Stoccoro et al., 2018). Within this scenario, an increase in mitochondrial DNA methylation and in the levels of DNMT3A were found in the spinal cord and muscles of an animal model of ALS (Maekawa et al., 2001). In agreement, an up-regulation of DNMT3A was also seen in postmortem mitochondrial fractions from the motor cortex of ALS patients (Chestnut et al., 2011). In addition, changes in 5mC and DNMT1 have also been noted in neuronal mitochondria from ALS patients (Chestnut et al., 2011). Given these findings, epigenetic modulation of mtDNA might also contribute to the pathogenesis of ALS and play a role in determining disease onset, as well as environmental vulnerability and response to toxicity (Iacobazzi et al., 2013).

Of note, an increase in mtDNA copy number has been reported in ALS patients, particularly in individuals with the *SOD1* and *C9orf72* mutations. However, subjects with the *SOD1* mutations also present a reduction in methylation levels in the D-loop region (Stoccoro et al., 2018). Since this region is critical for mtDNA replication and transcription, such demethylation might indicate a compensatory mechanism to counteract the overall upregulation of mtDNA (Stoccoro et al., 2018). Curiously, an increase in the mitochondrial gene responsible for the methylation of 16S rRNA was observed in spinal cord neurons and skeletal muscle of ALS transgenic mice (Wong et al., 2013).

Given the foregoing evidence, HDAC modulation, resulting in epigenetic modifications and consequent alterations of gene expression, mitochondrial function, and autophagy/mitophagy may exhibit a broad influence on neurogenesis. However, to date only a few studies (five at the date of revision of this article) have investigated the relationship among epigenetic modifications, mitochondrial function/bioenergetics, and neurogenesis. Nevertheless, one of these recently published studies has elegantly presented that cellular reprogramming by alterations in cellular metabolism is the main mechanism underlying changes in morphogenesis and that NSC differentiation can be modulated by mitochondrial function (Xie et al., 2019). Future studies are clearly necessary in order to elucidate the underpinnings of the relationship among epigenetics, mitochondrial function, and neurogenesis, and whether modulation of this relationship can alter the course of neurodegenerative diseases such as ALS.

## Conclusion

**FIGURE 2:**
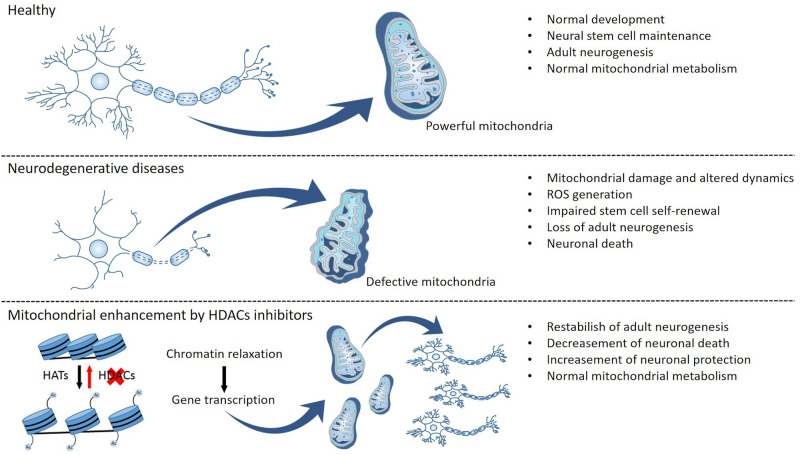
Mitochondrial function and epigenetic modulation as putative regulators of neurogenesis and neuronal survival. Functional mitochondria allow normal brain metabolism and development due to the maintenance of an endogenous neural stem cell pool and supporting the mechanisms of adult neurogenesis. Changes in mitochondrial function result in altered mitochondrial metabolism, dynamics, and transport, as well as generation of reactive oxygen species (ROS) and oxidative stress, and these disturbances can then culminate in the impairment of stem cell self-renewal, a decrease in adult neurogenesis, and neuronal death. Conversely, epigenetic modulation can promote mitochondrial metabolism, thus potentially reestablishing normal levels of adult neurogenesis while also promoting neuronal survival and preventing neuronal death.

Various lines of evidence primarily from preclinical studies performed in animal models of ALS suggest that preservation and/or an increase in mitochondrial function and metabolism could be beneficial in altering the course of this devastating neurodegenerative disease. In addition, increased mitochondrial function also has the potential to enhance adult neurogenesis, which is known to be altered by neurodegenerative processes. Thus, one postulates that improving mitochondrial function may promote NSC viability and proliferation, as well as neuronal differentiation and migration. As such, modulation of mitochondrial function may be an attractive beneficial strategy not only by promoting bioenergetics and reducing oxidative stress but also by facilitating pro-neurogenic processes in regions of the brain (and CNS) particularly affected by neurodegeneration NSCs differentiation can be modulated by mitochondrial function and/or responsible for the signs and symptoms of the disease (Figure 2). Future studies using postmortem brain tissue from patients afflicted with ALS are thus essential to further elucidate the relationship between mitochondrial (dys)function and neurogenesis in the ALS brain. Moreover, additional preclinical studies using *in vivo* ALS animal models are needed in order to determine whether therapeutic manipulations aimed at modulating mitochondrial function can impact not only neurogenic function but also disease severity and progression.

Finally, although progress has been made with regard to characterizing the contribution of epigenetics for the pathogenesis of ALS, the possibility that epigenetic modifications can indeed alter mitochondrial function and neurogenesis in ALS is still a matter of debate. Additional studies are clearly required in the near future to answer this challenging question and deepen our current understanding of ALS pathogenesis. Elucidating the relationships among epigenetics and mitochondrial function may not only provide the missing link in the understanding of the mechanisms underlying neurodegeneration in sporadic diseases such as ALS, but also might in turn open new doors in the search for effective disease-modifying treatments for these devastating neurological disorders.

## The Integrity of Research and Reporting

This is a review manuscript and therefore does not report original preclinical (i.e., animal) or clinical (i.e., human) data.

## Author Contributions

MC and TR drafted the initial version of the review. EH and AS helped with the formatting and figures. CB and JG-M collaborated in the revision of the version. All authors contributed to the article and approved the submitted version.

## Conflict of Interest

The authors declare that the research was conducted in the absence of any commercial or financial relationships that could be construed as a potential conflict of interest.

## References

[B1] Agostini M. Romeo F. Inoue S. Niklison-Chirou M. Elia A. Dinsdale D. (2016). Metabolic reprogramming during neuronal differentiation. *Cell Death Differ.* 23 1502–1514. 10.1038/cdd.2016.36 27058317 PMC5072427

[B2] Ajroud-Driss S. Siddique T. (2015). Sporadic and hereditary amyotrophic lateral sclerosis (ALS). *Biochim. Biophys. Acta* 1852 679–684. 10.1016/j.bbadis.2014.08.010 25193032

[B3] Alao J. P. (2007). The regulation of cyclin D1 degradation: roles in cancer development and the potential for therapeutic invention. *Mol. Cancer* 6:24. 10.1186/1476-4598-6-24 17407548 PMC1851974

[B4] Al-Chalabi A. Hardiman O. (2013). The epidemiology of ALS: a conspiracy of genes, environment and time. *Nat. Rev. Neurol.* 9 617–628. 10.1038/nrneurol.2013.203 24126629

[B5] Al-Chalabi A. Jones A. Troakes C. King A. Al-Sarraj S. van den Berg L. H. (2012). The genetics and neuropathology of amyotrophic lateral sclerosis. *Acta Neuropathol.* 124 339–352. 10.1007/s00401-012-1022-4 22903397

[B6] Almeida A. M. Bertoncini C. R. A. Borecky J. Souza-Pinto N. S. Vercesi A. E. (2006). Mitochondrial DNA damage associated with lipid peroxidation of the mitochondrial membrane induced by Fe2+-citrate. *An Acad. Bras Cienc.* 78 505–514. 10.1590/S0001-37652006000300010 16936939

[B7] Andreux P. A. Houtkooper R. H. Auwerx J. (2013). Pharmacological approaches to restore mitochondrial function. *Nat. Rev. Drug Discov.* 12 465–483. 10.1038/nrd4023 23666487 PMC3896945

[B8] Appleby-Mallinder C. Schaber E. Kirby J. Shaw P. J. Cooper-Knock J. Heath P. R. (2020). TDP43 proteinopathy is associated with aberrant DNA methylation in human amyotrophic lateral sclerosis. *Neuropathol. Appl. Neurobiol.* 10.1111/nan.12625 [Epub ahead of print]. 32365404

[B9] Aronica E. Baas F. Iyer A. Asbroek ten A. L. Morello G. Cavallaro S. (2015). Molecular classification of amyotrophic lateral sclerosis by unsupervised clustering of gene expression in motor cortex. *Neurobiol. Dis.* 74 359–376. 10.1016/j.nbd.2014.12.002 25500340

[B10] Bauer P. O. (2016). Methylation of C9orf72 expansion reduces RNA foci formation and dipeptide-repeat proteins expression in cells. *Neurosci. Lett.* 612 204–209. 10.1016/j.neulet.2015.12.018 26690922

[B11] Beeldman E. Raaphorst J. Klein Twennaar M. de Visser M. Schmand B. A. de Haan R. J. (2016). The cognitive profile of ALS: a systematic review and meta-analysis update. *J. Neurol. Neurosurg. Psychiatry* 87 611–619. 10.1136/jnnp-2015-310734 26283685

[B12] Bennett S. A. Tanaz R. Cobos S. N. Torrente M. P. (2019). Epigenetics in amyotrophic lateral sclerosis: a role for histone post-translational modifications in neurodegenerative disease. *Transl Res.* 204 19–30. 10.1016/j.trsl.2018.10.002 30391475 PMC6331271

[B13] Bernard-Marissal N. Chrast R. Schneider B. L. (2018). Endoplasmic reticulum and mitochondria in diseases of motor and sensory neurons: a broken relationship? *Cell Death Dis.* 9:333. 10.1038/s41419-017-0125-1 29491369 PMC5832431

[B14] Bernstein B. W. Bamburg J. R. (2003). Actin-ATP hydrolysis is a major energy drain for neurons. *J. Neurosci.* 23 1–6. 10.1523/JNEUROSCI.23-01-00001.2003 12514193 PMC6742122

[B15] Bertoncini C. R. A. Meneghini R. Galembeck F. Calió M. L. Carbonel A. F. Castro R. (2016). Preferential localization of iron in the chromatin of fe-enriched cells is linked to DNA cleavage sites and control of carcinogenesis. *J. Cancer Sci. Ther.* 8 213–215. 10.4172/1948-5956.1000415

[B16] Billups B. Forsythe I. D. (2002). Presynaptic mitochondrial calcium sequestration influences transmission at mammalian central synapses. *J. Neurosci.* 22 5840–5847. 10.1523/JNEUROSCI.22-14-05840.2002 12122046 PMC6757942

[B17] Bilsland L. G. Nirmalananthan N. Yip J. Greensmith L. Duchen M. R. (2008). Expression of mutant SOD1 in astrocytes induces functional deficits in motoneuron mitochondria. *J. Neurochem.* 107 1271–1283. 10.1111/j.1471-4159.2008.05699.x 18808448

[B18] Bilsland L. G. Sahai E. Kelly G. Golding M. Greensmith L. Schiavo G. (2010). Deficits in axonal transport precede ALS symptoms in vivo. *Proc. Natl. Acad. Sci. U.S.A.* 107 20523–20528. 10.1073/pnas.1006869107 21059924 PMC2996651

[B19] Bindokas V. P. Lee C. C. Colmers W. F. Miller R. J. (1998). Changes in mitochondrial function resulting from synaptic activity in the rat hippocampal slice. *J. Neurosci.* 18 4570–4587. 10.1523/JNEUROSCI.18-12-04570.1998 9614233 PMC6792701

[B20] Blair I. P. Williams K. L. Warraich S. T. Durnall J. C. Thoeng A. D. Manavis J. (2010). FUS mutations in amyotrophic lateral sclerosis: clinical, pathological, neurophysiological and genetic analysis. *J. Neurol. Neurosurg. Psychiatry* 81 639–645. 10.1136/jnnp.2009.194399 19965854

[B21] Boekhoorn K. Joels M. Lucassen P. J. (2006). Increased proliferation reflects glial and vascular-associated changes, but not neurogenesis in the presenile Alzheimer hippocampus. *Neurobiol. Dis.* 24 1–14. 10.1016/j.nbd.2006.04.017 16814555

[B22] Bonasio R. Tu S. Reinberg D. (2010). Molecular signals of epigenetic states. *Science* 330 612–616. 10.1126/science.1191078 21030644 PMC3772643

[B23] Bossers K. Wirz K. T. Meerhoff G. F. Essing A. H. van Dongen J. W. Houba P. (2010). Concerted changes in transcripts in the prefrontal cortex precede neuropathology in Alzheimer’s disease. *Brain* 133 3699–3723. 10.1093/brain/awq258 20889584

[B24] Bowling A. C. Schulz J. B. Brown R. H. Jr. Beal M. F. (1993). Superoxide dismutase activity, oxidative damage, and mitochondrial energy metabolism in familial and sporadic amyotrophic lateral sclerosis. *J. Neurochem.* 61 2322–2325. 10.1111/j.1471-4159.1993.tb07478.x 8245985

[B25] Bowman G. D. Poirier M. G. (2015). Post-translational modifications of histones that influence nucleosome dynamics. *Chem. Rev.* 115 2274–2295. 10.1021/cr500350x 25424540 PMC4375056

[B26] Bozzo F. Mirra A. Carri M. T. (2017). Oxidative stress and mitochondrial damage in the pathogenesis of ALS: new perspectives. *Neurosci. Let.* 636 3–8. 10.1016/j.neulet.2016.04.065 27150074

[B27] Brites D. Vaz A. R. (2014). Microglia centered pathogenesis in ALS: insights in cell interconnectivity. *Front. Cell Neurosci.* 8:117. 10.3389/fncel.2014.00117 24904276 PMC4033073

[B28] Brito M. D. da Silva G. F. G. Tilieri E. M. Araujo B. G. Calió M. L. Rosenstock T. R. (2019). Metabolic alteration and amyotrophic lateral sclerosis outcome: a systematic review. *Front. Neurol.* 10:1205. 10.3389/fneur.2019.01205 31824397 PMC6879457

[B29] Browne S. E. Yang L. DiMauro J. P. Fuller S. W. Licata S. C. Beal M. F. (2006). Bioenergetic abnormalities in discrete cerebral motor pathways presage spinal cord pathology in the G93A SOD1 mouse model of ALS. *Neurobiol. Dis.* 22 599–610. 10.1016/j.nbd.2006.01.001 16616851

[B30] Bruneteau G. Simonet T. Bauche S. Mandjee N. Malfatti E. Girard E. (2013). Muscle histone deacetylase 4 upregulation in amyotrophic lateral sclerosis: potential role in reinnervation ability and disease progression. *Brain* 136 2359–2368. 10.1093/brain/awt164 23824486

[B31] Bucchia M. Ramirez A. Parente V. Simone C. Nizzardo M. Magri F. (2015). Therapeutic development in amyotrophic lateral sclerosis. *Clin. Ther.* 37 668–680. 10.1016/j.clinthera.2014.12.020 25666449

[B32] Buratti E. Brindisi A. Giombi M. Tisminetzky S. Ayala Y. M. Baralle F. E. (2005). TDP-43 binds heterogeneous nuclear ribonucleoprotein A/B through its C-terminal tail: an important region for the inhibition of cystic fibrosis transmembrane conductance regulator exon 9 splicing. *J. Biol. Chem.* 280 37572–37584. 10.1074/jbc.M505557200 16157593

[B33] Butovsky O. Siddiqui S. Gabriely G. Lanser A. J. Dake B. Murugaiyan G. (2012). Modulating inflammatory monocytes with a unique microRNA gene signature ameliorates murine ALS. *J. Clin. Invest.* 122 3063–3087. 10.1172/JCI62636 22863620 PMC3428086

[B34] Cady J. Allred P. Bali T. Pestronk A. Goate A. Miller T. M. (2015). Amyotrophic lateral sclerosis onset is influenced by the burden of rare variants in known amyotrophic lateral sclerosis genes. *Ann. Neurol.* 77 100–113. 10.1002/ana.24306 25382069 PMC4293318

[B35] Cali T. Ottolini D. Brini M. (2012). Mitochondrial Ca(2+) and neurodegeneration. *Cell Calcium.* 52 73–85. 10.1016/j.ceca.2012.04.015 22608276 PMC3396847

[B36] Calingasan N. Y. Ho D. J. Wille E. J. Campagna M. V. Ruan J. Dumont M. (2008). Influence of mitochondrial enzyme deficiency on adult neurogenesis in mouse models of neurodegenerative diseases. *Neuroscience* 153 986–996. 10.1016/j.neuroscience.2008.02.071 18423880 PMC2907648

[B37] Calió M. L. Marinho D. S. Ko G. M. Rodrigues R. Carbonel A. F. Oyama L. M. (2014). Transplantation of bone marrow mesenchymal stem cells decreases superoxide, apoptosis and lipid peroxidation in brain of a spontaneously stroke model. *Free Radic. Biol. Med.* 70 141–154. 10.1016/j.freeradbiomed.2014.01.024 24525001

[B38] Camandola S. Mattson M. P. (2017). Brain metabolism in health, aging, and neurodegeneration. *EMBO J.* 36 1474–1492. 10.15252/embj.201695810 28438892 PMC5452017

[B39] Carri M. T. D’Ambrosi N. Cozzolino M. (2017). Pathways to mitochondrial dysfunction in ALS pathogenesis. *Biochem. Biophys. Res. Commun.* 483 1187–1193. 10.1016/j.bbrc.2016.07.055 27416757

[B40] Cha M. Y. Kim D. K. Mook-Jung I. (2015). The role of mitochondrial DNA mutation on neurodegenerative diseases. *Exp. Mol. Med.* 47:e150. 10.1038/emm.2014.122 25766619 PMC4351410

[B41] Chen H. Kankel M. W. Su S. C. Han S. W. S. Ofengeim D. (2018). Exploring the genetics and non-cell autonomous mechanisms underlying ALS/FTLD. *Cell Death Differ.* 25 646–660. 10.1038/s41418-018-0060-4 29459769 PMC5864209

[B42] Chen K. Bennett S. A. Rana N. Yousuf H. Said M. Taaseen S. (2018). Neurodegenerative disease proteinopathies are connected to distinct histone post-translational modification landscapes. *ACS Chem. Neurosci.* 9 838–848. 10.1021/acschemneuro.7b00297 29243911 PMC5906139

[B43] Chen Q. Nakajima A. Choi S. H. Xiong X. Sisodia S. S. Tang Y. P. (2008). Adult neurogenesis is functionally associated with AD-like neurodegeneration. *Neurobiol. Dis.* 29 316–326. 10.1016/j.nbd.2007.09.005 17980611 PMC2254142

[B44] Chen S. Zhang X. J. Li L. X. Wang Y. Zhong R. J. Le W. (2015). Histone deacetylase 6 delays motor neuron degeneration by ameliorating the autophagic flux defect in a transgenic mouse model of amyotrophic lateral sclerosis. *Neurosci. Bull.* 31 459–468. 10.1007/s12264-015-1539-3 26164555 PMC5563710

[B45] Chen X. Ye Y. Gu L. Sun J. Du Y. Liu W. J. (2019). H3K27me3 signal in the cis regulatory elements reveals the differentiation potential of progenitors during drosophila neuroglial development. *Genom. Proteom. Bioinform.* 17 297–304. 10.1016/j.gpb.2018.12.009 31195140 PMC6818177

[B46] Chestnut B. A. Chang Q. Price A. Lesuisse C. Wong M. Martin L. J. (2011). Epigenetic regulation of motor neuron cell death through DNA methylation. *J. Neurosci.* 31 16619–16636. 10.1523/JNEUROSCI.1639-11.2011 22090490 PMC3238138

[B47] Chi L. Gan L. Luo C. Lien L. Liu R. (2007). Temporal response of neural progenitor cells to disease onset and progression in amyotrophic lateral sclerosis-like transgenic mice. *Stem Cells Dev.* 16 579–588. 10.1089/scd.2006.0120 17784831

[B48] Chi L. Ke Y. Luo C. Li B. Gozal D. Kalyanaraman B. (2006). Motor neuron degeneration promotes neural progenitor cell proliferation, migration, and neurogenesis in the spinal cords of amyotrophic lateral sclerosis mice. *Stem Cells* 24 34–43. 10.1634/stemcells.2005-0076 16099995 PMC1828038

[B49] Chinnery P. F. Hudson G. (2013). Mitochondrial genetics. *Br. Med. Bull.* 106 135–159. 10.1093/bmb/ldt017 23704099 PMC3675899

[B50] Chio A. Borghero G. Pugliatti M. Ticca A. Calvo A. Moglia C. (2011). Large proportion of amyotrophic lateral sclerosis cases in Sardinia due to a single founder mutation of the TARDBP gene. *Arch. Neurol.* 68 594–598. 10.1001/archneurol.2010.352 21220647 PMC3513278

[B51] Chiu I. M. Morimoto E. T. A. Goodarzi H. Liao J. T. O’Keeffe S. Phatnani H. P. (2013). A neurodegeneration-specific gene-expression signature of acutely isolated microglia from an amyotrophic lateral sclerosis mouse model. *Cell Rep.* 4 385–401. 10.1016/j.celrep.2013.06.018 23850290 PMC4272581

[B52] Choi D. W. (1985). Glutamate neurotoxicity in cortical cell culture is calcium dependent. *Neurosci. Lett.* 58 293–297. 10.1016/0304-3940(85)90069-2 2413399

[B53] Collins M. Bowser R. (2017). “Chapter 4 – Molecular mechanisms of amyotrophic lateral sclerosis,” in *Molecular and Cellular Therapies for Motor Neuron Diseases* eds Boulis N. O’Connor D. Donsante A. (Elsevier: Academic Press), 61–99. 10.1016/B978-0-12-802257-3.00004-3

[B54] Coppedè F. Stoccoro A. Mosca L. Gallo R. Tarlarini C. Lunetta C. (2018). Increase in DNA methylation in patients with amyotrophic lateral sclerosis carriers of not fully penetrant SOD1 mutations. *Amyotroph. Lateral Scler. Frontotemporal Degener.* 19 93–101. 10.1080/21678421.2017.1367401 28859526

[B55] Corcoran L. J. Mitchison T. J. Liu Q. (2004). A novel action of histone deacetylase inhibitors in a protein aggresome disease model. *Curr. Biol.* 14 488–492. 10.1016/j.cub.2004.03.003 15043813

[B56] Cordeau-Lossouarn L. Vayssiere J. L. Larcher J. C. Gros F. Croizat B. (1991). Mitochondrial maturation during neuronal differentiation in vivo and in vitro. *Biol. Cell* 71 57–65. 10.1016/0248-4900(91)90051-n 1912948

[B57] Coussee E. De Smet P. Bogaert E. Elens I. Van Damme P. Willems P. (2011). G37R SOD1 mutant alters mitochondrial complex I activity, Ca2+ uptake and ATP production. *Cell Calcium* 494 217–225. 10.1016/j.ceca.2011.02.004 21388680

[B58] Cox L. E. Ferraiuolo L. Goodall E. F. Heath P. R. Higginbottom A. Mortiboys H. (2010). Mutations in CHMP2B in lower motor neuron predominant amyotrophic lateral sclerosis (ALS). *PLoS ONE* 5:e9872. 10.1371/journal.pone.0009872 20352044 PMC2844426

[B59] Cozzolino M. Pesaresi M. G. Amori I. Crosio C. Ferri A. Nencini M. (2009). Oligomerization of mutant SOD1 in mitochondria of motoneuronal cells drives mitochondrial damage and cell toxicity. *Antioxid. Redox Signal.* 11 1547–1558. 10.1089/ars.2009.2545 19344252

[B60] Crugnola V. Lamperti C. Lucchini V. Ronchi D. Peverelli L. Prelle A. (2010). Mitochondrial respiratory chain dysfunction in muscle from patients with amyotrophic lateral sclerosis. *Arch. Neurol.* 67 849–854. 10.1001/archneurol.2010.128 20625092

[B61] Cunniff B. McKenzie A. J. Heintz N. H. Howe A. K. (2016). AMPK activity regulates trafficking of mitochondria to the leading edge during cell migration and matrix invasion. *Mol. Biol. Cell.* 27 2662–2674. 10.1091/mbc.E16-05-0286 27385336 PMC5007087

[B62] Curtis M. A. Penney E. B. Pearson J. Dragunow M. Connor B. Faull R. L. (2005). The distribution of progenitor cells in the subependymal layer of the lateral ventricle in the normal and Huntington’s disease human brain. *Neuroscience* 132 777–788. 10.1016/j.neuroscience.2004.12.051 15837138

[B63] Dafinca R. Scaber J. Ababneh N. Lalic T. Weir G. Christian H. (2016). C9orf72 hexanucleotide expansions are associated with altered endoplasmic reticulum calcium homeostasis and stress granule formation in induced pluripotent stem cell-derived neurons from patients with amyotrophic lateral sclerosis and frontotemporal dementia. *Stem Cells* 34 2063–2078. 10.1002/stem.2388 27097283 PMC4979662

[B64] Damiano M. Starkov A. A. Petri S. Kipiani K. Kiaei M. Mattiazzi M. (2006). Neural mitochondrial Ca2+ capacity impairment precedes the onset of motor symptoms in G93A Cu/Zn-superoxide dismutase mutant mice. *J. Neurochem.* 96 1349–1361. 10.1111/j.1471-4159.2006.03619.x 16478527

[B65] David G. Barrett J. N. Barrett E. F. (1998). Evidence that mitochondria buffer physiological Ca2+ loads in lizard motor nerve terminals. *J. Physiol.* 509 59–65. 10.1111/j.1469-7793.1998.059bo.x 9547381 PMC2230953

[B66] Davis S. A. Itaman S. Khalid-Janney C. M. Sherard J. A. Dowell J. A. Cairns N. J. (2018). TDP-43 interacts with mitochondrial proteins critical for mitophagy and mitochondrial dynamics. *Neurosci. Lett.* 678 8–15. 10.1016/j.neulet.2018.04.053 29715546 PMC5975202

[B67] De Vos K. J. Chapman A. L. Tennant M. E. Manser C. Tudor E. L. Lau K. F. (2007). Familial amyotrophic lateral sclerosis-linked SOD1 mutants perturb fast axonal transport to reduce axonal mitochondria content. *Hum. Mol. Genet.* 16 2720–2728. 10.1093/hmg/ddm226 17725983 PMC4516806

[B68] Del Signore S. J. Amante D. J. Kim J. Stack E. C. Goodrich S. Cormier K. (2009). Combined riluzole and sodium phenylbutyrate therapy in transgenic amyotrophic lateral sclerosis mice. *Amyotroph Lateral Scler.* 10 85–94. 10.1080/17482960802226148 18618304

[B69] Deng J. Yang M. Chen Y. Chen X. Liu J. Sun S. (2015). FUS interacts with HSP60 to promote mitochondrial damage. *PLoS Genet.* 11:e1005357. 10.1371/journal.pgen.1005357 26335776 PMC4559378

[B70] Deng J. Wang P. Chen X. Cheng H. Liu J. Fushimi K. (2020). FUS interacts with ATP synthase beta subunit and induces mitochondrial unfolded protein response in cellular and animal models. *Proc. Natl. Acad. Sci. USA* 115, E9678–E9686. 10.1073/pnas.1806655115 30249657 PMC6187197

[B71] Desai M. Han G. Li T. Ross M. G. (2019). Programmed epigenetic DNA methylation-mediated reduced neuroprogenitor cell proliferation and differentiation in small-for-gestational-age offspring. *Neuroscience* 412 60–71. 10.1016/j.neuroscience.2019.05.044 31153962 PMC8034830

[B72] Devall M. Roubroeks J. Mill J. Weedon M. Lunnon K. (2016). Epigenetic regulation of mitochondrial function in neurodegenerative disease: new insights from advances in genomic technologies. *Neurosci. Lett.* 625 47–55. 10.1016/j.neulet.2016.02.013 26876477 PMC5747527

[B73] Di Giorgio F. P. Carrasco M. A. Siao M. C. Maniatis T. Eggan K. (2007). Non-cell autonomous effect of glia on motor neurons in an embryonic stem cell-based ALS model. *Nat. Neurosci.* 10 608–614. 10.1038/nn1885 17435754 PMC3139463

[B74] DiFebo F. Curti D. Botti F. Biella G. Bigini P. Mennini T. (2012). Neural precursors (NPCs) from adult L967Q mice display early commitment to “in vitro” neuronal differentiation and hyperexcitability. *Exp. Neurol.* 236 307–318. 10.1016/j.expneurol.2012.05.010 22634210

[B75] Douglas P. M. Dillin A. (2010). Protein homeostasis and aging in neurodegeneration. *J. Cell Biol.* 190 719–729. 10.1083/jcb.201005144 20819932 PMC2935559

[B76] Dupuis L. Gonzalez de Aguilar J. L. Oudart H. de Tapia M. Barbeito L. (2004). Mitochondria in amyotrophic lateral sclerosis: a trigger and a target. *Neurodegener Dis.* 1 245–254. 10.1159/000085063 16908975

[B77] Dupuis L. Pradat P. F. Ludolph A. C. Loeffler J. P. (2011). Energy metabolism in amyotrophic lateral sclerosis. *Lancet Neurol.* 10 75–82. 10.1016/S1474-4422(10)70224-6 21035400

[B78] Endo F. Komine O. Fujimori-Tonou N. Katsuno M. Jin S. Watanabe S. (2015). Astrocyte-derived TGF-β1 accelerates disease progression in ALS mice by interfering with the neuroprotective functions of microglia and T cells. *Cell Rep.* 11 592–604. 10.1016/j.celrep.2015.03.053 25892237

[B79] Fabricius C. Berthold C. H. Rydmark M. (1993). Axoplasmic organelles at nodes of ranvier. II. Occurrence and distribution in large myelinated spinal cord axons of the adult cat. *J. Neurocytol.* 22 941–954. 10.1007/bf01218352 7507976

[B80] Faissner A. Reinhard J. (2015). The extracellular matrix compartment of neural stem and glial progenitor cells. *Glia* 63 1330–1349. 10.1002/glia.22839 25913849

[B81] Fedele V. Roybon L. Nordstrom U. Li J. Y. Brundin P. (2011). Neurogenesis in the R6/2 mouse model of Huntington’s disease is impaired at the level of NeuroD1. *Neuroscience* 173 76–81. 10.1016/j.neuroscience.2010.08.022 20807561

[B82] Feng Y. Jankovic J. Wu Y. C. (2015). Epigenetic mechanisms in Parkinson’s disease. *J. Neurol. Sci.* 349 3–9. 10.1016/j.jns.2014.12.017 25553963

[B83] Ferraiuolo L. Higginbottom A. Heath P. R. Barber S. Greenald D. Kirby J. (2011). Dysregulation of astrocyte motoneuron cross-talk in mutant superoxide dismutase 1-related amyotrophic lateral sclerosis. *Brain* 134 2627–2641. 10.1093/brain/awr193 21908873 PMC3170534

[B84] Figueroa-Romero C. Guo K. Murdock B. J. Paez-Colasante X. Bassis C. M. Mikhail K. A. (2019). Temporal evolution of the microbiome, immune system and epigenome with disease progression in ALS mice. *Dis. Model Mech.* 13 dmm041947. 10.1242/dmm.041947 31597644 PMC6906635

[B85] Fiorito V. Chiabrando D. Tolosano E. (2018). Mitochondrial targeting in neurodegeneration: a heme perspective. *Pharmaceuticals (Basel)* 11:3. 10.3390/ph11030087 30231533 PMC6161291

[B86] Folmes C. D. Dzeja P. P. Nelson T. J. Terzic A. (2012). Metabolic plasticity in stem cell homeostasis and differentiation. *Cell Stem Cell.* 11 596–606. 10.1016/j.stem.2012.10.002 23122287 PMC3593051

[B87] Foran E. Bogush A. Goffredo M. Roncaglia P. Gustincich S. Pasinelli P. (2011). Motor neuron impairment mediated by a sumoylated fragment of the glial glutamate transporter EAAT2. *Glia* 59 1719–1731. 10.1002/glia.21218 21769946 PMC3896305

[B88] Frick P. Sellier C. Mackenzie I. R. A. Cheng C. Y. Tahraoui-Bories J. Martinat C. (2018). Novel antibodies reveal presynaptic localization of C9orf72 protein and reduced protein levels in C9orf72 mutation carriers. *Acta Neuropathol. Commun.* 6 72. 10.1186/s40478-018-0579-0 30075745 PMC6091050

[B89] Friedman R. C. Farh K. K. Burge C. B. Bartel D. P. (2009). Most mammalian mRNAs are conserved targets of microRNAs. *Genome Res.* 19 92–105. 10.1101/gr.082701.108 18955434 PMC2612969

[B90] Gage F. H. Temple S. (2013). Neural stem cells: generating and regenerating the brain. *Neuron* 80 588–601. 10.1016/j.neuron.2013.10.037 24183012

[B91] Gal J. Chen J. Barnett K. R. Yang L. Brumley E. Zhu H. (2013). HDAC6 regulates mutant SOD1 aggregation through two SMIR motifs and tubulin acetylation. *J. Biol. Chem.* 288 15035–15045. 10.1074/jbc.M112.431957 23580651 PMC3663524

[B92] Galán L. Gomez-Pinedo U. Guerrero A. Garcia-Verdugo J. M. Matias-Guiu J. (2017). Amyotrophic lateral sclerosis modifies progenitor neural proliferation in adult classic neurogenic brain niches. *BMC Neurol.* 17:173. 10.1186/s12883-017-0956-5 28874134 PMC5585932

[B93] Ganel R. Ho T. Maragakis N. J. Jackson M. Steiner J. P. Rothstein J. D. (2006). Selective up-regulation of the glial Na+-dependent glutamate transporter GLT1 by a neuroimmunophilin ligand results in neuroprotection. *Neurobiol. Dis.* 21 556–567. 10.1016/j.nbd.2005.08.014 16274998

[B94] Gao C. Chen X. Xu A. Cheng K. Shen J. (2018). Adaptor protein APPL2 affects adult antidepressant behaviors and hippocampal neurogenesis via regulating the sensitivity of glucocorticoid receptor. *Mol. Neurobiol.* 55 5537–5547. 10.1007/s12035-017-0785-y 28965332

[B95] Gerschutz A. Heinsen H. Grunblatt E. Wagner A. K. Bartl J. Meissner C. (2013). Neuron-specific mitochondrial DNA deletion levels in sporadic Alzheimer’s disease. *Curr. Alzheimer Res.* 10 1041–1046. 10.2174/15672050113106660166 24156256

[B96] Gershoni-Emek N. Altman T. Ionescu A. Costa C. J. Gradus-Pery T. Willis D. E. (2018). Localization of RNAi machinery to axonal branch points and growth cones is facilitated by mitochondria and is disrupted in ALS. *Front. Mol. Neurosci.* 11:311. 10.3389/fnmol.2018.00311 30233312 PMC6134038

[B97] Gijselinck I. Van Mossevelde S. van der Zee J. Sieben A. Engelborghs S. De Bleecker J. (2015). The C9orf72 repeat size correlates with onset age of disease, DNA methylation and transcriptional downregulation of the promoter. *Mol. Psychiatry* 21 1112–1124. 10.1038/mp.2015.159 26481318 PMC4960451

[B98] Giralt A. Villarroya F. (2012). SIRT3, a pivotal actor in mitochondrial functions: metabolism, cell death and aging. *Biochem. J.* 444 1–10. 10.1042/BJ20120030 22533670

[B99] Gjoneska E. Pfenning A. R. Mathys H. Quon G. Kundaje A. Tsai L.-H. (2015). Conserved epigenomic signals in mice and humans reveal immune basis of Alzheimer’s disease. *Nature* 518 365–369. 10.1038/nature14252 25693568 PMC4530583

[B100] Gonzalez de Aguilar J. L. Niederhauser-Wiederkehr C. Halter B. De Tapia M. Di Scala F. (2008). Gene profiling of skeletal muscle in an amyotrophic lateral sclerosis mouse model. *Physiol. Genom.* 32 207–218. 10.1152/physiolgenomics.00017.2007 18000159

[B101] Green D. R. Galluzzi L. Kroemer G. (2011). Mitochondria and the autophagy-inflammation-cell death axis in organismal aging. *Science* 333 1109–1112. 10.1126/science.1201940 21868666 PMC3405151

[B102] Haidet-Phillips A. M. Hester M. E. Miranda C. J. Meyer K. Braun L. Frakes A. (2011). Astrocytes from familial and sporadic ALS patients are toxic to motor neurons. *Nat. Biotechnol.* 29 824–828. 10.1038/nbt.1957 21832997 PMC3170425

[B103] Han S. M. Baig H. S. Hammarlund M. (2016). Mitochondria localize to injured axons to support regeneration. *Neuron* 92 1308–1323. 10.1016/j.neuron.2016.11.025 28009276 PMC5364819

[B104] Hayashi H. Kato S. (1989). Total manifestations of amyotrophic lateral sclerosis. ALS in the totally locked-in state. *J. Neurol. Sci.* 93 19–35. 10.1016/0022-510x(89)90158-5 2809628

[B105] Henry C. Pavese P. Blanc M. Labarere J. Leclercq P. Brion J. P. (2011). HIV infection and diabetes: experience and quality of life in patients with two chronic diseases. *Presse Med.* 40 e463–e470. 10.1016/j.lpm.2011.05.019 21831573

[B106] Herrero-Mendez A. Almeida A. Fernandez E. Maestre C. Moncada S. Bolanos J. P. (2009). The bioenergetic and antioxidant status of neurons is controlled by continuous degradation of a key glycolytic enzyme by APC/C-Cdh1. *Nat. Cell Biol.* 11 747–752. 10.1038/ncb1881 19448625

[B107] Higgins C. M. Jung C. Ding H. Xu Z. (2002). Mutant Cu, Zn superoxide dismutase that causes motoneuron degeneration is present in mitochondria in the CNS. *J. Neurosci.* 22:Rc215. 10.1523/JNEUROSCI.22-06-j0001.2002 11886899 PMC6758252

[B108] Hinnell C. Hurt C. S. Landau S. Brown R. G. Samuel M. (2012). Nonmotor versus motor symptoms: how much do they matter to health status in Parkinson’s disease? *Mov. Disord.* 27 236–241. 10.1002/mds.23961 21954027

[B109] Hollands C. Bartolotti N. Lazarov O. (2016). Alzheimer’s disease and hippocampal adult neurogenesis; exploring shared mechanisms. *Front. Neurosci.* 10:178. 10.3389/fnins.2016.00178 27199641 PMC4853383

[B110] Hsieh J. Nakashima K. Kuwabara T. Mejia E. Gage F. H. (2004). Histone deacetylase inhibition-mediated neuronal differentiation of multipotent adult neural progenitor cells. *Proc. Natl. Acad. Sci. U.S.A.* 10147 16659–16664. 10.1073/pnas.0407643101 15537713 PMC527137

[B111] Hwang J. Y. Aromolaran K. A. Zukin R. S. (2017). The emerging field of epigenetics in neurodegeneration and neuroprotection. *Nat. Rev. Neurosci.* 18 347–361. 10.1038/nrn.2017.46 28515491 PMC6380351

[B112] Iacobazzi V. Castegna A. Infantino V. Andria G. (2013). Mitochondrial DNA methylation as a next-generation biomarker and diagnostic tool. *Mol. Genet. Metab.* 110 25–34. 10.1016/j.ymgme.2013.07.012 23920043

[B113] Ilieva H. Polymenidou M. Cleveland D. W. (2009). Non-cell autonomous toxicity in neurodegenerative disorders: ALS and beyond. *J. Cell Biol.* 187 761–772. 10.1083/jcb.200908164 19951898 PMC2806318

[B114] Jaarsma D. Rognoni F. van Duijn W. Verspaget H. W. Haasdijk E. D. Holstege J. C. (2001). CuZn superoxide dismutase (SOD1) accumulates in vacuolated mitochondria in transgenic mice expressing amyotrophic lateral sclerosis-linked SOD1 mutations. *Acta Neuropathol.* 1024 293–305. 10.1007/s004010100399 11603803

[B115] Janssen C. Schmalbach S. Boeselt S. Sarlette A. Dengler R. Petri S. (2010). Differential histone deacetylase mRNA expression patterns in amyotrophic lateral sclerosis. *J. Neuropathol. Exp. Neurol.* 696 573–581. 10.1097/NEN.0b013e3181ddd404 20467334

[B116] Javaid N. Choi S. (2017). Acetylation- and methylation-related epigenetic proteins in the context of their targets. *Genes (Basel).* 8:E196. 10.3390/genes8080196 28783137 PMC5575660

[B117] Ji A. L. Zhang X. Chen W. W. Huang W. J. (2017). Genetics insight into the amyotrophic lateral sclerosis/frontotemporal dementia spectrum. *J. Med. Genet.* 54 145–154. 10.1136/jmedgenet-2016-104271 28087719

[B118] Jimenez-Pacheco A. Franco J. M. Lopez S. Gomez-Zumaquero J. M. Magdalena Leal-Lasarte M. Caballero-Hernandez D. E. (2017). Epigenetic mechanisms of gene regulation in amyotrophic lateral sclerosis. *Adv. Exp. Med. Biol.* 978 255–275. 10.1007/978-3-319-53889-1_14 28523551

[B119] Jin K. Peel A. L. Mao X. O. Xie L. Cottrell B. A. Henshall D. C. (2004). Increased hippocampal neurogenesis in Alzheimer’s disease. *Proc. Natl. Acad. Sci. U.S.A.* 1011 343–347. 10.1073/pnas.2634794100 14660786 PMC314187

[B120] Johann S. Heitzer M. Kanagaratnam M. Goswami A. Rizo T. Weis J. (2015). NLRP3 inflammasome is expressed by astrocytes in the SOD1 mouse model of ALS and in human sporadic ALS patients. *Glia* 63 2260–2273. 10.1002/glia.22891 26200799

[B121] Joshi A. U. Saw N. L. Vogel H. Cunnigham A. D. Shamloo M. Mochly-Rosen D. (2018). Inhibition of Drp1/Fis1 interaction slows progression of amyotrophic lateral sclerosis. *EMBO Mol. Med.* 10:e8166. 10.15252/emmm.201708166 29335339 PMC5840540

[B122] Jung C. Higgins C. M. Xu Z. (2002). Mitochondrial electron transport chain complex dysfunction in a transgenic mouse model for amyotrophic lateral sclerosis. *J. Neurochem.* 833 535–545. 10.1046/j.1471-4159.2002.01112.x 12390515

[B123] Jury N. Abarzua S. Diaz I. Guerra M. V. Ampuero E. Cubillos P. (2020). Widespread loss of the silencing epigenetic mark H3K9me3 in astrocytes and neurons along with hippocampal-dependent cognitive impairment in C9orf72 BAC transgenic mice. *Clin. Epigenet.* 12:32. 10.1186/s13148-020-0816-9 32070418 PMC7029485

[B124] Kameda T. Imamura T. Nakashima K. (2018). Epigenetic regulation of neural stem cell differentiation towards spinal cord regeneration. *Cell Tissue Res.* 3711 189–199. 10.1007/s00441-017-2656-2 28695279

[B125] Kang S. H. Li Y. Fukaya M. Lorenzini I. Cleveland D. W. Ostrow L. W. (2013). Degeneration and impaired regeneration of gray matter oligodendrocytes in amyotrophic lateral sclerosis. *Nat. Neurosci.* 16 571–579. 10.1038/nn.3357 23542689 PMC3637847

[B126] Kann O. Kovacs R. Heinemann U. (2003). Metabotropic receptor-mediated Ca2+signaling elevates mitochondrial Ca2+ and stimulates oxidative metabolism in hippocampal slice cultures. *J. Neurophysiol.* 90 613–621. 10.1152/jn.00042.2003 12724360

[B127] Karki P. Smith K. Johnson J. Jr. Aschner M. Lee E. Y. (2015). Genetic dys-regulation of astrocytic glutamate transporter EAAT2 and its implications in neurological disorders and manganese toxicity. *Neurochem. Res.* 40 380–388. 10.1007/s11064-014-1391-2 25064045 PMC4308576

[B128] Kawahara Y. Mieda-Sato A. (2012). TDP-43 promotes microRNA biogenesis as a component of the Drosha and Dicer complexes. *Proc. Natl. Acad. Sci. U.S.A.* 109 3347–3352. 10.1073/pnas.1112427109 22323604 PMC3295278

[B129] Kawamata H. Manfredi G. (2010). Mitochondrial dysfunction and intracellular calcium dysregulation in ALS. *Mech. Ageing Dev.* 131 517–526. 10.1016/j.mad.2010.05.003 20493207 PMC2933290

[B130] Keogh M. J. Chinnery P. F. (2015). Mitochondrial DNA mutations in neurodegeneration. *Biochim. Biophys. Acta* 184711 1401–1411. 10.1016/j.bbabio.2015.05.015 26014345

[B131] Khalil B. Lievens J. C. (2017). Mitochondrial quality control in amyotrophic lateral sclerosis: towards a common pathway? *Neural Regen. Res.* 127 1052–1061. 10.4103/1673-5374.211179 28852382 PMC5558479

[B132] Kim D. Nguyen M. D. Dobbin M. M. Fischer A. Sananbenesi F. Rodgers J. T. (2007). SIRT1 deacetylase protects against neurodegeneration in models for alzheimer’s disease and amyotrophic lateral sclerosis. *EMBO J.* 26 3169–3179. 10.1038/sj.emboj.7601758 17581637 PMC1914106

[B133] Kim S. H. Shanware N. P. Bowler M. J. Tibbetts R. S. (2010). Amyotrophic lateral sclerosis-associated proteins TDP-43 and FUS/TLS function in a common biochemical complex to co-regulate HDAC6 mRNA. *J. Biol. Chem.* 285 34097–34105. 10.1074/jbc.M110.154831 20720006 PMC2962508

[B134] Kirby D. M. Rennie K. J. Smulders-Srinivasan T. K. Acin-Perez R. Whittington M. Enriquez J. A. (2009). Transmitochondrial embryonic stem cells containing pathogenic mtDNA mutations are compromised in neuronal differentiation. *Cell Prolif.* 424 413–424. 10.1111/j.1365-2184.2009.00612.x 19552636 PMC2730481

[B135] Kodavati M. Wang H. Hegde M. L. (2020). Altered mitochondrial dynamics in motor neuron disease: an emerging perspective. *Cells* 9:E1065. 10.3390/cells9041065 32344665 PMC7226538

[B136] Koentjoro B. Park J. S. Sue C. M. (2017). Nix restores mitophagy and mitochondrial function to protect against PINK1/Parkin-related Parkinson’s disease. *Sci. Rep.* 7:44373. 10.1038/srep44373 28281653 PMC5345073

[B137] Koh H. Kim H. Kim M. J. Park J. Lee H. J. Chung J. (2012). Silent information regulator 2 (Sir2) and Forkhead box O (FOXO) complement mitochondrial dysfunction and dopaminergic neuron loss in Drosophila PTEN-induced kinase 1 (PINK1) null mutant. *J. Biol. Chem.* 287 12750–12758. 10.1074/jbc.M111.337907 22378780 PMC3339960

[B138] Kohl Z. Regensburger M. Aigner R. Kandasamy M. Winner B. Aigner L. (2010). Impaired adult olfactory bulb neurogenesis in the R6/2 mouse model of Huntington’s disease. *BMC Neurosci.* 11:114. 10.1186/1471-2202-11-114 20836877 PMC2945356

[B139] König H. G. Coughlan K. S. Kinsella S. Breen B. A. Prehn J. H. (2014). The BCL-2 family protein Bid is critical for proinflammatory signaling in astrocytes. *Neurobiol. Dis.* 70 99–107. 10.1016/j.nbd.2014.06.008 24956542

[B140] Koreman E. Sun X. Lu Q. R. (2018). Chromatin remodeling and epigenetic regulation of oligodendrocyte myelination and myelin repair. *Mol. Cell Neurosci.* 87 18–26. 10.1016/j.mcn.2017.11.010 29254827 PMC5828965

[B141] Koval E. D. Shaner C. Zhang P. du Maine X. Fischer K. Tay J. (2013). Method for widespread microRNA-155 inhibition prolongs survival in ALS-model mice. *Hum. Mol. Genet.* 22 4127–4135. 10.1093/hmg/ddt261 23740943 PMC3781640

[B142] Krasemann S. Madore C. Cialic R. Baufeld C. Calcagno N. El Fatimy R. (2017). The TREM2-APOE pathway drives the transcriptional phenotype of dysfunctional microglia in neurodegenerative diseases. *Immunity* 47 566–581.e9. 10.1016/j.immuni.2017.08.008 28930663 PMC5719893

[B143] Kunst C. B. (2004). Complex genetics of amyotrophic lateral sclerosis. *Am. J. Hum. Genet.* 75 933–947. 10.1086/426001 15478096 PMC1182156

[B144] Lagali P. S. Picketts D. J. (2011). Matters of life and death: the role of chromatin remodeling proteins in retinal neuron survival. *J. Ocul. Biol. Dis. Inform.* 43 111–120. 10.1007/s12177-012-9080-3 23289056 PMC3382293

[B145] Lapucci A. Cavone L. Buonvicino D. Felici R. Gerace E. Zwergel C. (2017). Effect of class II HDAC inhibition on glutamate transporter expression and survival in SOD1-ALS mice. *Neurosci. Lett.* 656 120–125. 10.1016/j.neulet.2017.07.033 28732762

[B146] Le Grand J. N. Gonzalez-Cano L. Pavlou M. A. Schwamborn J. C. (2015). Neural stem cells in Parkinson’s disease: a role for neurogenesis defects in onset and progression. *Cell Mol. Life Sci.* 724 773–797. 10.1007/s00018-014-1774-1 25403878 PMC11113294

[B147] Lee J. Hyeon S. J. Im H. Ryu H. Kim Y. Ryu H. (2016). Astrocytes and microglia as non-cell autonomous players in the pathogenesis of ALS. *Exp. Neurobiol.* 25 233–240. 10.5607/en.2016.25.5.233 27790057 PMC5081469

[B148] Lee J. C. Shin J. H. Park B. W. Kim G. S. Kim J. C. Kang K. S. (2011). Region-specific changes in the immunoreactivity of SIRT1 expression in the central nervous system of SOD1G93A transgenic mice as an in vivo model of amyotrophic lateral sclerosis. *Brain Res.* 1433 20–28. 10.1016/j.brainres.2011.11.019 22137654

[B149] Lee J. Y. Koga H. Kawaguchi Y. Tang W. Wong E. Gao Y. S. (2010). HDAC6 controls autophagosome maturation essential for ubiquitin-selective quality-control autophagy. *EMBO J.* 29 969–980. 10.1038/emboj.2009.405 20075865 PMC2837169

[B150] Levy M. Faas G. C. Saggau P. Craigen W. J. Sweatt J. D. (2003). Mitochondrial regulation of synaptic plasticity in the hippocampus. *J. Biol. Chem.* 278 17727–17734. 10.1074/jbc.M212878200 12604600

[B151] Li R. Strykowski R. Meyer M. Mulcrone P. Krakora D. Suzuki M. (2012). Male-specific differences in proliferation, neurogenesis, and sensitivity to oxidative stress in neural progenitor cells derived from a rat model of ALS. *PLoS ONE* 711:e48581. 10.1371/journal.pone.0048581 23144905 PMC3493558

[B152] Li X. L. Shu S. Li X. G. Liu Q. Liu F. Cui B. (2016). CHCHD10 is not a frequent causative gene in Chinese ALS patients. *Amyotroph. Lateral Scler. Frontotemporal Degener.* 17 458–460. 10.3109/21678421.2016.1170151 27077676

[B153] Li Z. Okamoto K. Hayashi Y. Sheng M. (2004). The importance of dendritic mitochondria in the morphogenesis and plasticity of spines and synapses. *Cell* 119 873–887. PMID:NOPMID 15607982 10.1016/j.cell.2004.11.003

[B154] Liang Q. Benavides G. A. Vasilopoulos A. Gius D. Darley-Usmar V. Zhang J. (2013). Bioenergetic and autophagic control by Sirt3 in response to nutrient deprivation in mouse embryonic fibroblasts. *Biochem. J.* 454 249–257. 10.1042/BJ20130414 23767918 PMC3927421

[B155] Lin-Hendel E. G. McManus M. J. Wallace D. C. Anderson S. A. Golden J. A. (2016). Differential mitochondrial requirements for radially and non-radially migrating cortical neurons: implications for mitochondrial disorders. *Cell Rep.* 15 229–237. 10.1016/j.celrep.2016.03.024 27050514 PMC5412722

[B156] Liu W. Yamashita T. Tian F. Morimoto N. Ikeda Y. Deguchi K. (2013). Mitochondrial fusion and fission proteins expression dynamically change in a murine model of amyotrophic lateral sclerosis. *Curr. Neurovasc. Res.* 10 222–230. 10.2174/15672026113109990060 23713734

[B157] Liu H. Song N. (2016). Molecular mechanism of adult neurogenesis and its association with human brain diseases. *J. Cent. Nerv. Syst. Dis.* 8, 5–11. 10.4137/JCNSD.S32204 27375363 PMC4915785

[B158] Liu Z. Martin L. J. (2006). The adult neural stem, and progenitor cell niche is altered in amyotrophic lateral sclerosis mouse brain. *J. Compar. Neurol.* 497 468–488. 10.1002/cne.21012 16736475

[B159] Lombard D. B. Alt F. W. Cheng H. L. Bunkenborg J. Streeper R. S. Mostoslavsky R. (2007). Mammalian Sir2 homolog SIRT3 regulates global mitochondrial lysine acetylation. *Mol. Cell. Biol.* 27 8807–8814. 10.1128/MCB.01636-07 17923681 PMC2169418

[B160] Lopez-Gonzalez R. Lu Y. Gendron T. F. Karydas A. Tran H. Yang D. (2016). Poly(GR) in C9ORF72-Related ALS/FTD compromises mitochondrial function and increases oxidative stress and DNA damage in iPSC-derived motor neurons. *Neuron* 92 383–391. 10.1016/j.neuron.2016.09.015 27720481 PMC5111366

[B161] Lovejoy D. B. Guillemin G. J. (2014). The potential for transition metal-mediated neurodegeneration in amyotrophic lateral sclerosis. *Front. Aging Neurosci.* 6:173. 10.3389/fnagi.2014.00173 25100994 PMC4107949

[B162] Ludolph A. C. Brettschneider J. Weishaupt J. H. (2012). Amyotrophic lateral sclerosis. *Curr. Opin. Neurol.* 25 530–535. 10.1097/WCO.0b013e328356d328 22918486

[B163] Maekawa M. Sugano K. Ushiama M. Fukayama N. Nomoto K. Kashiwabara H. (2001). Heterogeneity of DNA methylation status analyzed by bisulfite-PCR-SSCP and correlation with clinico-pathological characteristics in colorectal cancer. *Clin. Chem. Lab. Med.* 39 121–128. 10.1515/CCLM.2001.021 11341745

[B164] Magrane J. Hervias I. Henning M. S. Damiano M. Kawamata H. Manfredi G. (2009). Mutant SOD1 in neuronal mitochondria causes toxicity and mitochondrial dynamics abnormalities. *Hum. Mol. Genet.* 1823 4552–4564. 10.1093/hmg/ddp421 19779023 PMC2773270

[B165] Magrane J. Sahawneh M. A. Przedborski S. Estevez A. G. Manfredi G. (2012). Mitochondrial dynamics and bioenergetic dysfunction is associated with synaptic alterations in mutant SOD1 motor neurons. *J. Neurosci.* 321 229–242. 10.1523/jneurosci.1233-11.2012 22219285 PMC3566782

[B166] Majounie E. Renton A. E. Mok K. Dopper E. G. Waite A. Rollinson S. (2012). Frequency of the C9orf72 hexanucleotide repeat expansion in patients with amyotrophic lateral sclerosis and frontotemporal dementia: a cross-sectional study. *Lancet Neurol.* 11 323–330. 10.1016/S1474-4422(12)70043-1 22406228 PMC3322422

[B167] Malik R. Meng H. Wongkongkathep P. Corrales C. I. Sepanj N. Atlasi R. S. (2019). The molecular tweezer CLR01 inhibits aberrant superoxide dismutase 1 (SOD1) self-assembly in vitro and in the G93A-SOD1 mouse model of ALS. *J. Biol. Chem.* 294 3501–3513. 10.1074/jbc.RA118.005940 30602569 PMC6416427

[B168] Mancuso R. Del Valle J. Morell M. Pallas M. Osta R. Navarro X. (2014). Lack of synergistic effect of resveratrol and sigma-1 receptor agonist PRE-084 in SOD1G93A ALS mice: overlapping effects or limited therapeutic opportunity? *Orphanet. J. Rare. Dis.* 9:78. 10.1186/1750-1172-9-78 24885036 PMC4035830

[B169] Marcuzzo S. Kapetis D. Mantegazza R. Baggi F. Bonanno S. Barzago C. (2014). Altered miRNA expression is associated with neuronal fate in G93A-SOD1 ependymal stem progenitor cells. *Exp. Neurol.* 253 91–101. 10.1016/j.expneurol.2013.12.007 24365539

[B170] Marsh S. E. Blurton-Jones M. (2017). Neural stem cell therapy for neurodegenerative disorders: the role of neurotrophic support. *Neurochem. Int.* 106 94–100. 10.1016/j.neuint.2017.02.006 28219641 PMC5446923

[B171] Martin D. Thompson M. A. Nadler J. V. (1993). The neuroprotectivea agent riluzole inhibits release of glutamate and aspartate from slices of hippocampal area CA1. *Eur. J. Pharmacol.* 250 473–476. 10.1016/0014-2999(93)90037-I 8112408

[B172] Martinez-Merino L. Iridoy M. Galbete A. Roldan M. Rivero A. Acha B. (2018). Evaluation of chitotriosidase and CC-chemokine ligand 18 as biomarkers of microglia activation in amyotrophic lateral sclerosis. *Neurodegener. Dis.* 184 208–215. 10.1159/000490920 30134252

[B173] Marxreiter F. Regensburger M. Winkler J. (2013). Adult neurogenesis in Parkinson’s disease. *Cell. Mol. Life Sci.* 703 459–473. 10.1007/s00018-012-1062-x 22766974 PMC11113680

[B174] Masala A. Sanna S. Esposito S. Rassu M. Galioto M. Zinellu A. (2018). Epigenetic changes associated with the expression of amyotrophic lateral sclerosis (ALS) causing genes. *Neuroscience* 390 1–11. 10.1016/j.neuroscience.2018.08.009 30134203

[B175] Matias-Guiu J. A. Cabrera-Martin M. N. Oreja-Guevara C. Carreras J. L. Matias-Guiu J. (2016). Pittsburgh compound B and other amyloid positron emission tomography tracers for the study of white matter and multiple sclerosis. *Ann. Neurol.* 801:166. 10.1002/ana.24666 27098362

[B176] Mattson M. P. Gleichmann M. Cheng A. (2008). Mitochondria in neuroplasticity and neurological disorders. *Neuron* 60 748–766. 10.1016/j.neuron.2008.10.010 19081372 PMC2692277

[B177] Mehler M. F. (2008). Epigenetics and the nervous system. *Ann. Neurol.* 64 602–617. 10.1002/ana.21595 19107999

[B178] Merson T. D. Dixon M. P. Collin C. Rietze R. L. Bartlett P. F. Thomas T. (2006). The transcriptional coactivator querkopf controls adult neurogenesis. *J. Neurosci.* 2644 11359–11370. 10.1523/jneurosci.2247-06.2006 17079664 PMC6674553

[B179] Mirochnic S. Wolf S. Staufenbiel M. Kempermann G. (2009). Age effects on the regulation of adult hippocampal neurogenesis by physical activity and environmental enrichment in the APP23 mouse model of Alzheimer disease. *Hippocampus* 1910 1008–1018. 10.1002/hipo.20560 19219917

[B180] Morahan J. M. Yu B. Trent R. J. Pamphlett R. (2007). Are metallothionein genes silenced in ALS? *Toxicol. Lett.* 1681 83–87. 10.1016/j.toxlet.2006.11.003 17156946

[B181] Morahan J. M. Yu B. Trent R. J. Pamphlett R. (2009). A genome-wide analysis of brain DNA methylation identifies new candidate genes for sporadic amyotrophic lateral sclerosis. *Amyotroph. Lateral Scler.* 10 418–429. 10.3109/17482960802635397 19922134

[B182] Mu Y. Lee S. W. Gage F. H. (2010). Signaling in adult neurogenesis. *Curr. Opin. Neurobiol.* 204 416–423. 10.1016/j.conb.2010.04.010 20471243 PMC2942965

[B183] Murphy M. P. (2009). How mitochondria produce reactive oxygen species. *Biochem. J.* 4171 1–13. 10.1042/bj20081386 19061483 PMC2605959

[B184] Nagai M. Re D. B. Nagata T. Chalazonitis A. Jessell T. M. Wichterle H. (2007). Astrocytes expressing ALS-linked mutated SOD1 release factors selectively toxic to motor neurons. *Nat. Neurosci.* 10 615–622. 10.1038/nn1876 17435755 PMC3799799

[B185] Naia L. Ferreira I. L. Cunha-Oliveira T. Duarte A. I. Ribeiro M. Rosenstock T. R. (2014). Activation of IGF-1 and insulin signaling pathways ameliorate mitochondrial function and energy metabolism in Huntington’s Disease human lymphoblasts. *Mol. Neurobiol.* 511 331–348. 10.1007/s12035-014-8735-4 24841383

[B186] Naia L. Rosenstock T. R. Oliveira A. M. Oliveira-Sousa S. I. Caldeira G. L. Carmo C. (2017). Comparative mitochondrial-based protective effects of resveratrol and nicotinamide in huntington’s disease models. *Mol. Neurobiol.* 54 5385–5399. 10.1007/s12035-016-0048-3 27590140

[B187] Nikodemova M. Small A. L. Smith S. M. Mitchell G. S. Watters J. J. (2014). Spinal but not cortical microglia acquire an atypical phenotype with high VEGF, galectin-3 and osteopontin, and blunted inflammatory responses in ALS rats. *Neurobiol. Dis.* 69 43–53. 10.1016/j.nbd.2013.11.009 24269728 PMC4079765

[B188] Nissanka N. Moraes C. T. (2018). Mitochondrial DNA damage and reactive oxygen species in neurodegenerative disease. *FEBS Lett.* 5925 728–742. 10.1002/1873-3468.12956 29281123 PMC6942696

[B189] Nonneman A. Criem N. Lewandowski S. A. Nuyts R. Thal D. R. Pfrieger F. W. (2018). Astrocyte-derived Jagged-1 mitigates deleterious Notch signaling in amyotrophic lateral sclerosis. *Neurobiol. Dis.* 119 26–40. 10.1016/j.nbd.2018.07.012 30010003

[B190] Nunnari J. Suomalainen A. (2012). Mitochondria: in sickness and in health. *Cell.* 1486 1145–1159. 10.1016/j.cell.2012.02.035 22424226 PMC5381524

[B191] Oates N. Pamphlett R. (2007). An epigenetic analysis of SOD1 and VEGF in ALS. *Amyotroph. Lateral Scler.* 82 83–86. 10.1080/17482960601149160 17453634

[B192] Oh M. Choi I. K. Kwon H. J. (2008). Inhibition of histone deacetylase1 induces autophagy. *Biochem. Biophys. Res. Commun.* 369 1179–1183. 10.1016/j.bbrc.2008.03.019 18342621

[B193] Olzmann J. A. Li L. Chin L. S. (2008). Aggresome formation and neurodegenerative diseases: therapeutic implications. *Curr. Med. Chem.* 15 47–60. 10.2174/092986708783330692 18220762 PMC4403008

[B194] Onesto E. Colombrita C. Gumina V. Borghi M. O. Dusi S. Doretti A. (2016). Gene-specific mitochondria dysfunctions in human TARDBP and C9ORF72 fibroblasts. *Acta Neuropathol. Commun.* 4:47. 10.1186/s40478-016-0316-5 27151080 PMC4858818

[B195] Paez-Colasante X. Figueroa-Romero C. Sakowski S. A. Goutman S. A. Feldman E. L. (2015). Amyotrophic lateral sclerosis: mechanisms and therapeutics in the epigenomic era. *Nat. Rev. Neurol.* 115 266–279. 10.1038/nrneurol.2015.57 25896087

[B196] Palomo G. M. Granatiero V. Kawamata H. Konrad C. Kim M. Arreguin A. J. (2018). Parkin is a disease modifier in the mutant SOD1 mouse model of ALS. *EMBO Mol. Med.* 10:e8888. 10.15252/emmm.201808888 30126943 PMC6180298

[B197] Pansarasa O. Bordoni M. Drufuca L. Diamanti L. Sproviero D. Trotti R. (2018). Lymphoblastoid cell lines as a model to understand amyotrophic lateral sclerosis disease mechanisms. *Dis. Model. Mech.* 11:dmm031625. 10.1242/dmm.031625 29419416 PMC5897724

[B198] Pardo A. C. Wong V. Benson L. M. Dykes M. Tanaka K. Rothstein J. D. (2006). Loss of the astrocyte glutamate transporter GLT1 modifies disease in SOD1(G93A) mice. *Exp. Neurol.* 201 120–130. 10.1016/j.expneurol.2006.03.028 16753145

[B199] Parisi C. Arisi I. D’Ambrosi N. Storti A. E. Brandi R. D’Onofrio M. (2013). Dysregulated microRNAs in amyotrophic lateral sclerosis microglia modulate genes linked to neuroinflammation. *Cell Death Dis.* 4:e959. 10.1038/cddis.2013.491 24336079 PMC3877562

[B200] Parisi C. Napoli G. Amadio S. Spalloni A. Apolloni S. Longone P. (2016). MicroRNA-125b regulates microglia activation and motor neuron death in ALS. *Cell Death Differ.* 23 531–541. 10.1038/cdd.2015.153 26794445 PMC5072447

[B201] Parkinson G. M. Dayas C. V. Smith D. W. (2014). Increased mitochondrial DNA deletions in substantia nigra dopamine neurons of the aged rat. *Curr. Aging Sci.* 73 155–160. 10.2174/1874609808666150122150850 25612740

[B202] Pasinelli P. Belford M. E. Lennon N. Bacskai B. J. Hyman B. T. Trotti D. (2004). Amyotrophic lateral sclerosis-associated SOD1 mutant proteins bind and aggregate with Bcl-2 in spinal cord mitochondria. *Neuron* 431 19–30. 10.1016/j.neuron.2004.06.021 15233914

[B203] Pasinetti G. M. Bilski A. E. Zhao W. (2013). Sirtuins as therapeutic targets of ALS. *Cell Res.* 239 1073–1074. 10.1038/cr.2013.94 23856645 PMC3760621

[B204] Pelizzoni I. Macco R. Zacchetti D. Grohovaz F. Codazzi F. (2008). Iron and calcium in the central nervous system: a close relationship in health and sickness. *Biochem. Soc. Trans.* 36 (Pt 6), 1309–1312. 10.1042/bst0361309 19021546

[B205] Peng Q. Masuda N. Jiang M. Li Q. Zhao M. Ross C. A. (2008). The antidepressant sertraline improves the phenotype, promotes neurogenesis and increases BDNF levels in the R6/2 Huntington’s disease mouse model. *Exp. Neurol.* 2101 154–163. 10.1016/j.expneurol.2007.10.015 18096160 PMC2278120

[B206] Perera N. D. Turner B. J. (2016). AMPK signalling and defective energy metabolism in amyotrophic lateral sclerosis. *Neurochem. Res.* 41 544–553. 10.1007/s11064-015-1665-3 26202426

[B207] Perier C. Bender A. Garcia-Arumi E. Melia M. J. Bove J. Laub C. (2013). Accumulation of mitochondrial DNA deletions within dopaminergic neurons triggers neuroprotective mechanisms. *Brain* 136 (Pt 8), 2369–2378. 10.1093/brain/awt196 23884809

[B208] Petanjek Z. Judaš M. Šimic G. Rasin M. R. Uylings H. B. Rakic P. (2011). Extraordinary neoteny of synaptic spines in the human prefrontal cortex. *Proc. Natl. Acad. Sci. U.S.A.* 108 13281–13286. 10.1073/pnas.1105108108 21788513 PMC3156171

[B209] Pinto M. Moraes C. T. (2014). Mitochondrial genome changes and neurodegenerative diseases. *Biochim. Biophys. Acta* 18428 1198–1207. 10.1016/j.bbadis.2013.11.012 24252612 PMC4283582

[B210] Probst A. V. Dunleavy E. Almouzni G. (2009). Epigenetic inheritance during the cell cycle. *Nat. Rev. Mol. Cell Biol.* 103 192–206. 10.1038/nrm2640 19234478

[B211] Prozorovski T. Schulze-Topphoff U. Glumm R. Baumgart J. Schroter F. Ninnemann O. (2008). Sirt1 contributes critically to the redox-dependent fate of neural progenitors. *Nat. Cell Biol.* 104 385–394. 10.1038/ncb1700 18344989

[B212] Rafalski V. A. Brunet A. (2011). Energy metabolism in adult neural stem cell fate. *Prog. Neurobiol.* 932 182–203. 10.1016/j.pneurobio.2010.10.007 21056618

[B213] Ramasamy S. Narayanan G. Sankaran S. Yu Y. H. Ahmed S. (2013). Neural stem cell survival factors. *Arch. Biochem. Biophys.* 534 71–87. 10.1016/j.abb.2013.02.004 23470250

[B214] Ravera S. Bonifacino T. Bartolucci M. Milanese M. Gallia E. Provenzano F. (2018). Characterization of the mitochondrial aerobic metabolism in the pre- and perisynaptic districts of the SOD1G93A mouse model of amyotrophic lateral sclerosis. *Mol. Neurobiol.* 5512 9220–9233. 10.1007/s12035-018-1059-z 29656361

[B215] Re D. B. Le Verche V. Yu C. Amoroso M. W. Politi K. A. Phani S. (2014). Necroptosis drives motor neuron death in models of both sporadic and familial ALS. *Neuron* 81 1001–1008. 10.1016/j.neuron.2014.01.011 24508385 PMC3951532

[B216] Requejo-Aguilar R. Lopez-Fabuel I. Fernandez E. Martins L. M. Almeida A. Bolanos J. P. (2014). PINK1 deficiency sustains cell proliferation by reprogramming glucose metabolism through HIF1. *Nat. Commun.* 5:4514. 10.1038/ncomms5514 25058378

[B217] Rodriguez J. J. Verkhratsky A. (2011a). Neurogenesis in Alzheimer’s disease. *J. Anat.* 2191 78–89. 10.1111/j.1469-7580.2011.01343.x 21323664 PMC3130162

[B218] Rodriguez J. J. Verkhratsky A. (2011b). Neuroglial roots of neurodegenerative diseases? *Mol. Neurobiol.* 43 87–96. 10.1007/s12035-010-8157-x 21161612

[B219] Rosenstock T. R. (2013). Lysine K-deacetylase inhibitors: the real next step to neuropsychiatric and neurodegenerative disorders? *Biohelikon: Cell Biol.* 2:a8.

[B220] Rosenstock T. R. Bertoncini C. R. Teles A. V. Hirata H. Fernandes M. J. Smaili S. S. (2010a). Glutamate-induced alterations in Ca2+ signaling are modulated by mitochondrial Ca2+ handling capacity in brain slices of R6/1 transgenic mice. *Eur. J. Neurosci.* 321 60–70. 10.1111/j.1460-9568.2010.07268.x 20608968

[B221] Rosenstock T. R. Duarte A. I. Rego A. C. (2010b). Mitochondrial-associated metabolic changes and neurodegeneration in Huntington’s disease – From clinical features to the bench. *Curr. Drug. Targets* 1110 1218–1236. 10.2174/1389450111007011218 20840066

[B222] Rosenstock T. R. Carvalho A. C. Jurkiewicz A. Frussa-Filho R. Smaili S. S. (2004). Mitochondrial calcium, oxidative stress and apoptosis in a neurodegenerative disease model induced by 3-nitropropionic acid. *J. Neurochem.* 885 1220–1228. 10.1046/j.1471-4159.2003.02250.x 15009678

[B223] Rossaert E. Pollari E. Jaspers T. Van Helleputte L. Jarpe M. Van Damme P. (2019). Restoration of histone acetylation ameliorates disease and metabolic abnormalities in a FUS mouse model. *Acta Neuropathol. Commun.* 7:107. 10.1186/s40478-019-0750-2 31277703 PMC6612190

[B224] Rothstein J. D. Patel S. Regan M. R. Haenggeli C. Huang Y. H. Bergles D. E. (2005). Betalactam antibiotics offer neuroprotection by increasing glutamate transporter expression. *Nature* 433 73–77. 10.1038/nature03180 15635412

[B225] Rothstein J. D. Van Kammen M. Levey A. I. Martin L. J. Kuncl R. W. (1995). Selective loss of glial glutamate transporter GLT-1 in amyotrophic lateral sclerosis. *Ann. Neurol.* 38 73–84. 10.1002/ana.410380114 7611729

[B226] Rouaux C. Jokic N. Mbebi C. Boutillier S. Loeffler J. P. Boutillier A. L. (2003). Critical loss of CBP/p300 histone acetylase activity by caspase-6 during neurodegeneration. *EMBO. J.* 2224 6537–6549. 10.1093/emboj/cdg615 14657026 PMC291810

[B227] Rutishauser U. (2008). Polysialic acid in the plasticity of the developing and adult vertebrate nervous system. *Nat. Rev. Neurosci.* 9 26–35. 10.1038/nrn2285 18059411

[B228] Rutishauser U. Landmesser L. (1996). Polysialic acid in the vertebrate nervous system: a promoter of plasticity in cell-cell interactions. *Trends Neurosci.* 19 422–427. 10.1016/0166-2236(96)10041-2 8888519

[B229] Sabatelli M. Eusebi F. Al-Chalabi A. Conte A. Madia F. Luigetti M. (2009). Rare missense variants of neuronal nicotinic acetylcholine receptor altering receptor function are associated with sporadic amyotrophic lateral sclerosis. *Hum. Mol. Genet.* 18 3997–4006. 10.1093/hmg/ddp339 19628475

[B230] Sainath R. Ketschek A. Grandi L. Gallo G. (2017). CSPGs inhibit axon branching by impairing mitochondria-dependent regulation of actin dynamics and axonal translation. *Dev. Neurobiol.* 77 454–473. 10.1002/dneu.22420 27429169 PMC5243930

[B231] Salvatori I. Valle C. Ferri A. Carri M. T. (2017). SIRT3 and mitochondrial metabolism in neurodegenerative diseases. *Neurochem. Int.* 109 184–192. 10.1016/j.neuint.2017.04.012 28449871

[B232] Sarkar S. Malovic E. Harischandra D. S. Ngwa H. A. Ghosh A. Hogan C. (2018). Manganese exposure induces neuroinflammation by impairing mitochondrial dynamics in astrocytes. *Neurotoxicology* 64 204–218. 10.1016/j.neuro.2017.05.009 28539244 PMC5698176

[B233] Sasaki S. Horie Y. Iwata M. (2007). Mitochondrial alterations in dorsal root ganglion cells in sporadic amyotrophic lateral sclerosis. *Acta Neuropathol.* 114 633–639. 10.1007/s00401-007-0299-1 17929040

[B234] Sasaki S. Iwata M. (1996). Impairment of fast axonal transport in the proximal axons of anterior horn neurons in amyotrophic lateral sclerosis. *Neurology* 472 535–540. 10.1212/WNL.47.2.535 8757033

[B235] Sawada H. (2017). Clinical efficacy of ederavone for the treatment of amyotrophic lateral sclerosis. *Expert Opin. Pharmacother.* 18 735–738. 10.1080/14656566.2017.1319937 28406335

[B236] Schiaffino L. Bonafede R. Scambi I. Parrella E. Pizzi M. Mariotti R. (2018). Acetylation state of RelA modulated by epigenetic drugs prolongs survival and induces a neuroprotective effect on ALS murine model. *Sci. Rep.* 81:12875. 10.1038/s41598-018-30659-4 30150770 PMC6110772

[B237] Schinder A. F. Olson E. C. Spitzer N. C. Montal M. (1996). Mitochondrial dysfunction is a primary event in glutamate neurotoxicity. *J. Neurosci.* 16 6125–6133. 10.1523/JNEUROSCI.16-19-06125.1996 8815895 PMC6579180

[B238] Schmalbach S. Petri S. (2010). Histone deacetylation and motor neuron degeneration. *CNS Neurol. Disord. Drug. Targets* 93 279–284. 10.2174/187152710791292684 20406183

[B239] Seetharaman S. V. Prudencio M. Karch C. Holloway S. P. Borchelt D. R. Hart P. J. (2009). Immature copper-zinc superoxide dismutase and familial amyotrophic lateral sclerosis. *Exp. Biol. Med. (Maywood)* 234 1140–1154. 10.3181/0903-MR-104 19596823 PMC2850267

[B240] Sheng Z. H. (2017). The Interplay of axonal energy homeostasis and mitochondrial trafficking and anchoring. *Trends Cell. Biol.* 27 403–416. 10.1016/j.tcb.2017.01.005 28228333 PMC5440189

[B241] Sheng Z. H. Cai Q. (2012). Mitochondrial transport in neurons: impact on synaptic homeostasis and neurodegeneration. *Nat. Rev. Neurosci.* 132 77–93. 10.1038/nrn3156 22218207 PMC4962561

[B242] Shi P. Wei Y. Zhang J. Gal J. Zhu H. (2010). Mitochondrial dysfunction is a converging point of multiple pathological pathways in amyotrophic lateral sclerosis. *J. Alzheimers. Dis.* 20 S311–S324. 10.3233/jad-2010-100366 20463400

[B243] Shneyer B. I. Ušaj M. Wiesel-Motiuk N. Regev R. Henn A. (2017). ROS induced distribution of mitochondria to filopodia by Myo19 depends on a class specific tryptophan in the motor domain. *Sci. Rep.* 7:11577. 10.1038/s41598-017-11002-9 28912530 PMC5599611

[B244] Sidlauskaite E. Gibson J. W. Megson I. L. Whitfield P. D. Tovmasyan A. Batinic-Haberle I. (2018). Mitochondrial ROS cause motor deficits induced by synaptic inactivity: implications for synapse pruning. *Redox. Biol.* 16 344–351. 10.1016/j.redox.2018.03.012 29587245 PMC5953219

[B245] Siklos L. Engelhardt J. Harati Y. Smith R. G. Joo F. Appel S. H. (1996). Ultrastructural evidence for altered calcium in motor nerve terminals in amyotropic lateral sclerosis. *Ann. Neurol.* 392 203–216. 10.1002/ana.410390210 8967752

[B246] Simpson J. M. Gil-Mohapel J. Pouladi M. A. Ghilan M. Xie Y. Hayden M. R. (2011). Altered adult hippocampal neurogenesis in the YAC128 transgenic mouse model of Huntington disease. *Neurobiol. Dis.* 412 249–260. 10.1016/j.nbd.2010.09.012 20875859

[B247] Simuni T. Sethi K. (2008). Nonmotor manifestations of Parkinson’s disease. *Ann. Neurol.* 64 S65–S80. 10.1002/ana.21472 19127582

[B248] Smith E. F. Shaw P. J. De Vos K. J. (2019). The role of mitochondria in amyotrophic lateral sclerosis. *Neurosci. Lett.* 710:132933. 10.1016/j.neulet.2017.06.052 28669745

[B249] Smith G. M. Gallo G. (2018). The role of mitochondria in axon development and regeneration. *Dev. Neurobiol.* 78 221–237. 10.1002/dneu.22546 29030922 PMC5816701

[B250] Song L. Chen L. Zhang X. Li J. Le W. (2014). Resveratrol ameliorates motor neuron degeneration and improves survival in SOD1G93A mouse model of amyotrophic lateral sclerosis. *Biomed. Res. Int.* 2014:483501. 10.1155/2014/483501 25057490 PMC4095711

[B251] Song W. Song Y. Kincaid B. Bossy B. Bossy-Wetzel E. (2013). Mutant SOD1G93A triggers mitochondrial fragmentation in spinal cord motor neurons: neuroprotection by SIRT3 and PGC-1α. *Neurobiol. Dis.* 51 72–81. 10.1016/j.nbd.2012.07.004 22819776 PMC3992938

[B252] Sotelo-Silveira J. R. Lepanto P. Elizondo V. Horjales S. Palacios F. Martinez-Palma L. (2009). Axonal mitochondrial clusters containing mutant SOD1 in transgenic models of ALS. *Antioxid. Redox. Signal.* 117 1535–1545. 10.1089/ars.2009.2614 19344250 PMC2842590

[B253] Srinivasan K. Friedman B. A. Larson J. L. Lauffer B. E. Goldstein L. D. Appling L. L. (2016). Untangling the brain’s neuroinflammatory and neurodegenerative transcriptional responses. *Nat Commun.* 7:11295. 10.1038/ncomms11295 27097852 PMC4844685

[B254] Stoccoro A. Mosca L. Carnicelli V. Cavallari U. Lunetta C. Marocchi A. (2018). Mitochondrial DNA copy number and D-loop region methylation in carriers of amyotrophic lateral sclerosis gene mutations. *Epigenomics* 10 1431–1443. 10.2217/epi-2018-0072 30088417

[B255] Stout J. C. Paulsen J. S. Queller S. Solomon A. C. Whitlock K. B. Campbell J. C. (2011). Neurocognitive signs in prodromal huntington disease. *Neuropsychology* 251 1–14. 10.1037/a0020937 20919768 PMC3017660

[B256] Taes I. Timmers M. Hersmus N. Bento-Abreu A. Van Den Bosch L. Van Damme P. (2013). Hdac6 deletion delays disease progression in the SOD1G93A mouse model of ALS. *Hum. Mol. Genet.* 22 1783–1790. 10.1093/hmg/ddt028 23364049

[B257] Tafuri F. Ronchi D. Magri F. Comi G. P. Corti S. (2015). SOD1 misplacing and mitochondrial dysfunction in amyotrophic lateral sclerosis pathogenesis. *Front. Cell Neurosci.* 9:336. 10.3389/fncel.2015.00336 26379505 PMC4548205

[B258] Tang T. S. Slow E. Lupu V. Stavrovskaya I. G. Sugimori M. Llinas R. (2005). Disturbed Ca2+ signaling and apoptosis of medium spiny neurons in Huntington’s disease. *Proc. Natl. Acad. Sci. U.S.A.* 1027 2602–2607. 10.1073/pnas.0409402102 15695335 PMC548984

[B259] Tang Y. Zucker R. S. (1997). Mitochondrial involvement in post-tetanic potentiation of synaptic transmission. *Neuron* 18 483–491. 10.1016/S0896-6273(00)81248-9 9115741

[B260] Thompson A. Boekhoorn K. Van Dam A. M. Lucassen P. J. (2008). Changes in adult neurogenesis in neurodegenerative diseases: cause or consequence? *Genes Brain Behav.* 7 28–42. 10.1111/j.1601-183X.2007.00379.x 18184368

[B261] Tibshirani M. Tradewell M. L. Mattina K. R. Minotti S. Yang W. Zhou H. (2015). Cytoplasmic sequestration of FUS/TLS associated with ALS alters histone marks through loss of nuclear protein arginine methyltransferase 1. *Hum. Mol. Genet.* 24 773–786. 10.1093/hmg/ddu494 25274782 PMC4291251

[B262] Tradewell M. L. Yu Z. Tibshirani M. Boulanger M. C. Durham H. D. Richard S. (2012). Arginine methylation by PRMT1 regulates nuclear-cytoplasmic localization and toxicity of FUS/TLS harbouring ALS-linked mutations. *Hum. Mol. Genet.* 21 136–149. 10.1093/hmg/ddr448 21965298

[B263] Tremolizzo L. Messina P. Conti E. Sala G. Cecchi M. Airoldi L. (2014). Whole-blood global DNA methylation is increased in amyotrophic lateral sclerosis independently of age of onset. *Amyotroph Lateral Scler Frontotemporal Degener.* 15 98–105. 10.3109/21678421.2013.851247 24224837

[B264] Trüe O. Matthias P. (2012). Interplay between histone deacetylases and autophagy–from cancer therapy to neurodegeneration. *Immunol. Cell. Biol.* 90 78–84. 10.1038/icb.2011.103 22124372

[B265] Valle C. Salvatori I. Gerbino V. Rossi S. Palamiuc L. Rene F. (2014). Tissue-specific deregulation of selected HDACs characterizes ALS progression in mouse models: pharmacological characterization of SIRT1 and SIRT2 pathways. *Cell Death. Dis.* 5 e1296. 10.1038/cddis.2014.247 24946089 PMC4611720

[B266] van den Berge S. A. van Strien M. E. Korecka J. A. Dijkstra A. A. Sluijs J. A. Kooijman L. (2011). The proliferative capacity of the subventricular zone is maintained in the parkinsonian brain. *Brain* 134 (Pt 11), 3249–3263. 10.1093/brain/awr256 22075520

[B267] van Rheenen W. Shatunov A. Dekker A. M. McLaughlin R. L. Diekstra F. P. Pulit S. L. (2016). Genome-wide association analyses identify new risk variants and the genetic architecture of amyotrophic lateral sclerosis. *Nat Genet.* 48 1043–1048. 10.1038/ng.3622 27455348 PMC5556360

[B268] Vande Velde C. Dion P. A. Rouleau G. A. (2011). Amyotrophic lateral sclerosis: new genes, new models, and new mechanisms. *F1000 Biol. Rep.* 3:18. 10.3410/b3-18 21941597 PMC3169903

[B269] Vandoorne T. De Bock K. Van Den Bosch L. (2018). Energy metabolism in ALS: an underappreciated opportunity? *Acta. Neuropathol.* 1354 489–509. 10.1007/s00401-018-1835-x 29549424 PMC5978930

[B270] Vayssiere J. L. Cordeau-Lossouarn L. Larcher J. C. Basseville M. Gros F. Croizat B. (1992). Participation of the mitochondrial genome in the differentiation of neuroblastoma cells. *In Vitro Cell. Dev. Biol.* 28a 763–772. 10.1007/bf02631065 1483966

[B271] Verstreken P. Ly C. V. Venken K. J. Koh T. W. Zhou Y. Bellen H. J. (2005). Synaptic mitochondria are critical for mobilization of reserve pool vesicles at Drosophila neuromuscular junctions. *Neuron* 47 365–378. 10.1016/j.neuron.2005.06.018 16055061

[B272] Vijayvergiya C. Beal M. F. Buck J. Manfredi G. (2005). Mutant superoxide dismutase 1 forms aggregates in the brain mitochondrial matrix of amyotrophic lateral sclerosis mice. *J. Neurosci.* 2510 2463–2470. 10.1523/jneurosci.4385-04.2005 15758154 PMC6725162

[B273] Wang W. Li L. Lin W. L. Dickson D. W. Petrucelli L. Zhang T. (2013). The ALS disease-associated mutant TDP-43 impairs mitochondrial dynamics and function in motor neurons. *Hum. Mol. Genet* 22 4706–4719. 10.1093/hmg/ddt319 23827948 PMC3820133

[B274] Wang W. Wang L. Lu J. Siedlak S. L. Fujioka H. Liang J. (2016). The inhibition of TDP-43 mitochondrial localization blocks its neuronal toxicity. *Nat. Med.* 22 869–878. 10.1038/nm.4130 27348499 PMC4974139

[B275] Wang X. Arai S. Song X. Reichart D. Du K. Pascual G. (2008). Induced ncRNAs allosterically modify RNA-binding proteins in cis to inhibit transcription. *Nature* 454 126–130. 10.1038/nature06992 18509338 PMC2823488

[B276] Warita H. Hayashi T. Murakami T. Manabe Y. Abe K. (2001). Oxidative damage to mitochondrial DNA in spinal motoneurons of transgenic ALS mice. *Brain. Res. Mol. Brain. Res.* 89 147–152. 10.1016/s0169-328x(01)00029-8 11311985

[B277] Webster C. P. Smith E. F. Bauer C. S. Moller A. Hautbergue G. M. Ferraiuolo L. (2016). The C9orf72 protein interacts with Rab1a and the ULK1 complex to regulate initiation of autophagy. *EMBO J.* 35 1656–1676. 10.15252/embj.201694401 27334615 PMC4969571

[B278] Webster C. P. Smith E. F. Grierson A. J. De Vos K. J. (2018). C9orf72 plays a central role in Rab GTPase-dependent regulation of autophagy. *Small GTPases* 95 399–408. 10.1080/21541248.2016.1240495 27768524 PMC5997165

[B279] Wiedemann F. R. Manfredi G. Mawrin C. Beal M. F. Schon E. A. (2002). Mitochondrial DNA and respiratory chain function in spinal cords of ALS patients. *J. Neurochem.* 804 616–625. 10.1046/j.0022-3042.2001.00731.x 11841569

[B280] Williamson T. L. Cleveland D. W. (1999). Slowing of axonal transport is a very early event in the toxicity of ALS-linked SOD1 mutants to motor neurons. *Nat. Neurosci.* 21 50–56. 10.1038/4553 10195180

[B281] Winner B. Couillard-Despres S. Geyer M. Aigner R. Bogdahn U. Aigner L. (2008). Dopaminergic lesion enhances growth factor-induced striatal neuroblast migration. *J. Neuropathol. Exp. Neurol.* 672 105–116. 10.1097/nen.0b013e3181630cff 18219258

[B282] Winner B. Winkler J. (2015). Adult neurogenesis in neurodegenerative diseases. *Cold Spring Harb. Perspect. Biol.* 74:a021287. 10.1101/cshperspect.a021287 25833845 PMC4382734

[B283] Wong M. Gertz B. Chestnut B. A. Martin L. J. (2013). Mitochondrial DNMT3A and DNA methylation in skeletal muscle and CNS of transgenic mouse models of ALS. *Front. Cell Neurosci.* 7:279. 10.3389/fncel.2013.00279 24399935 PMC3872319

[B284] Wu X. Chen P. S. Dallas S. Wilson B. Block M. L. Wang C. C. (2008). Histone deacetylase inhibitors up-regulate astrocyte GDNF and BDNF gene transcription and protect dopaminergic neurons. *Int. J. Neuropsychopharmacol.* 11 1123–1134. 10.1017/S1461145708009024 18611290 PMC2579941

[B285] Xavier J. M. Morgado A. L. Sola S. Rodrigues C. M. (2013). Mitochondrial translocation of p53 modulates neuronal fate by preventing differentiation-induced mitochondrial stress. *Antioxid. Redox. Signal.* 217 1009–1024. 10.1089/ars.2013.5417 24329038 PMC4123470

[B286] Xie K. Ngo S. Rong J. Sheppard A. (2019). Modulation of mitochondrial respiration underpins neuronal differentiation enhanced by lutein. *Neural Regen. Res.* 14 87–99. 10.4103/1673-5374.243713 30531082 PMC6262990

[B287] Xiong Y. Guan K. L. (2012). Mechanistic insights into the regulation of metabolic enzymes by acetylation. *J. Cell. Biol.* 1982 155–164. 10.1083/jcb.201202056 22826120 PMC3410420

[B288] Yamanaka K. Chun S. J. Boillee S. Fujimori-Tonou N. Yamashita H. Gutmann D. H. (2008). Astrocytes as determinants of disease progression in inherited amyotrophic lateral sclerosis. *Nat. Neurosci.* 11 251–253. 10.1038/nn2047 18246065 PMC3137510

[B289] Yang F. He X.-P. Russell J. Lu B. (2003). Ca2+influx-independent synaptic potentiation mediated by mitochondrial Na+-Ca2+exchanger and protein kinase C. *J. Cell Biol.* 163 511–523. 10.1083/jcb.200307027 14610054 PMC2173636

[B290] Yang Y. Gozen O. Vidensky S. Robinson M. B. Rothstein J. D. (2010). Epigenetic regulation of neuron-dependent induction of astroglial synaptic protein GLT1. *Glia* 58 277–286. 10.1002/glia.20922 19672971 PMC2794958

[B291] Yao T. Deng Z. Gao Y. Sun J. Kong X. Huang Y. (2016). Ire1alpha in pomc neurons is required for thermogenesis and glycemia. *Diabetes* 663 663–673. 10.2337/db16-0533 28028078 PMC5319716

[B292] Yoo Y. E. Ko C. P. (2011). Treatment with trichostatin A initiated after disease onset delays disease progression and increases survival in a mouse model of amyotrophic lateral sclerosis. *Exp. Neuro.* 231 147–159. 10.1016/j.expneurol.2011.06.003 21712032

[B293] Young P. E. Kum J. S. Buckland M. E. Pamphlett R. Suter C. M. (2017). Epigenetic differences between monozygotic twins discordant for amyotrophic lateral sclerosis (ALS) provide clues to disease pathogenesis. *PLoS ONE* 12:e0182638. 10.1371/journal.pone.0182638 28797086 PMC5552194

[B294] Yu I. T. Park J. Y. Kim S. H. Lee J. S. Kim Y. S. Son H. (2009). Valproic acid promotes neuronal differentiation by induction of proneural factors in association with H4 acetylation. *Neuropharmacology* 562 473–480. 10.1016/j.neuropharm.2008.09.019 19007798

[B295] Zarei S. Carr K. Reiley L. Diaz K. Guerra O. Altamirano P. F. (2015). A comprehensive review of amyotrophic lateral sclerosis. *Surg. Neurol. Int.* 6:171. 10.4103/2152-7806.169561 26629397 PMC4653353

[B296] Zenisek D. Matthews G. (2000). The role of mitochondria in presynaptic calcium handling at a ribbon synapse. *Neuron* 25 229–237. 10.1016/S0896-6273(00)80885-5 10707986

[B297] Zhang C. L. Ho P. L. Kintner D. B. Sun D. Chiu S. Y. (2010). Activity-dependent regulation of mitochondrial motility by calcium and Na/K-ATPase at nodes of Ranvier of myelinated nerves. *J. Neurosci.* 30 3555–3566. 10.1523/JNEUROSCI.4551-09.2010 20219989 PMC3548432

[B298] Zhao C. Deng W. Gage F. H. (2008). Mechanisms and functional implications of adult neurogenesis. *Cell* 1324 645–660. 10.1016/j.cell.2008.01.033 18295581

[B299] Zhou J. Yi J. Fu R. Liu E. Siddique T. Rios E. (2010). Hyperactive intracellular calcium signaling associated with localized mitochondrial defects in skeletal muscle of an animal model of amyotrophic lateral sclerosis. *J. Biol. Chem.* 2851 705–712. 10.1074/jbc.m109.041319 19889637 PMC2804218

